# Immune responses to CCAR1 and other dermatomyositis autoantigens are associated with attenuated cancer emergence

**DOI:** 10.1172/JCI150201

**Published:** 2022-01-18

**Authors:** David F. Fiorentino, Christopher A. Mecoli, Matthew C. Rosen, Lorinda S. Chung, Lisa Christopher-Stine, Antony Rosen, Livia Casciola-Rosen

**Affiliations:** 1 Department of Dermatology, Stanford University School of Medicine, Redwood City, California, USA.; 2 Division of Rheumatology, Johns Hopkins University School of Medicine, Baltimore, Maryland, USA.; 3 Department of Neurobiology, The University of Chicago, Chicago, Illinois, USA.; 4 Division of Immunology and Rheumatology, Stanford University School of Medicine, Palo Alto, California, USA.

**Keywords:** Autoimmunity, Immunology, Antigen, Autoimmune diseases, Cancer

## Abstract

**BACKGROUND:**

The temporal clustering of a cancer diagnosis with dermatomyositis (DM) onset is strikingly associated with autoantibodies against transcriptional intermediary factor 1-γ (TIF1-γ). Nevertheless, many patients with anti–TIF1-γ antibodies never develop cancer. We investigated whether additional autoantibodies are found in anti–TIF1-γ–positive patients without cancer.

**METHODS:**

Using a proteomic approach, we defined 10 previously undescribed autoantibody specificities in 5 index anti–TIF1-γ–positive DM patients without cancer. These were subsequently examined in discovery (*n =* 110) and validation (*n =* 142) cohorts of DM patients with anti–TIF1-γ autoantibodies.

**RESULTS:**

We identified 10 potentially novel autoantibodies in anti–TIF1-γ–positive DM patients, 6 with frequencies ranging from 3% to 32% in 2 independent DM cohorts. Autoantibodies recognizing cell division cycle and apoptosis regulator protein 1 (CCAR1) were the most frequent, and were significantly negatively associated with contemporaneous cancer (discovery cohort OR 0.27 [95% CI 0.7–1.00], *P =* 0.050; validation cohort OR 0.13 [95% CI 0.03–0.59], *P =* 0.008). When cancer did emerge, it occurred significantly later in anti-CCAR1–positive compared with anti-CCAR1–negative patients (median time from DM onset 4.3 vs. 0.85 years, respectively; *P =* 0.006). Cancers that emerged were more likely to be localized (89% of anti-CCAR1–positive cancers presenting at stage 0 or 1 compared with 42% of patients without anti-CCAR1 antibodies, *P =* 0.02). As the number of additional autoantibody specificities increased in anti–TIF1-γ–positive DM patients, the frequency of cancer decreased (*P <* 0.001).

**CONCLUSION:**

As the diversity of immune responses in anti–TIF1-γ DM patients increases, the likelihood of cancer emerging decreases. Our findings have important relevance for cancer risk stratification in DM patients and for understanding natural immune regulation of cancer in humans.

**TRIAL REGISTRATION:**

Not applicable.

**FUNDING SOURCES:**

The NIH, the Donald B. and Dorothy L. Stabler Foundation, and the Huayi and Siuling Zhang Discovery Fund.

## Graphical abstract

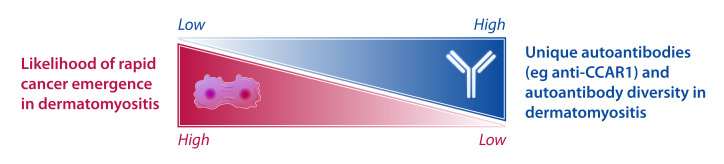

## Introduction

A temporal clustering of cancer and dermatomyositis (DM) in a subgroup of DM patients has been appreciated for decades, with diagnosis of cancers particularly prominent in the –3- to +3-year window around DM onset (termed cancer-associated myositis, or CAM; refs. 1–3). Recent studies have demonstrated that CAM is much more likely to occur in association with specific autoantibodies, with a majority of cancers occurring in those patients with autoantibodies recognizing tripartite motif–containing 33 (TRIM33), also known as transcriptional intermediary factor 1-γ (TIF1-γ) (4, 5). There is additional heterogeneity within this anti–TIF1-γ–positive group, and several subgroups are apparent: (a) patients who develop a cancer within 1 year of DM onset, (b) patients who do not manifest with cancer until more than 1 year after DM diagnosis, and (c) patients who never develop a cancer (6–9). Studies of anticancer immunity and response to checkpoint blockade suggest that baseline breadth of the immune response correlates broadly with successful cancer control (10, 11). Defining additional autoantibody specificities that are enriched in anti–TIF1-γ–positive DM patients in whom cancer does not emerge could therefore provide insights into the mechanisms underlying the cancer-DM association and inform cancer screening strategies in the clinic.

Recent findings in systemic sclerosis (SSc) show that autoantibodies against the catalytic subunit of RNA polymerase III (POLR3) define a subgroup of SSc with an increased incidence of, and close temporal relationship to, cancer (12, 13). In anti-POLR3–positive SSc patients, somatic mutations and loss of heterozygosity (LOH) at the POLR3A locus in the associated cancers were frequent; such genetic changes were not seen in cancers from patients with other immune responses in SSc (14). These somatic mutations appear to drive an immune response to the mutated epitope that spreads to the wild-type antigen (14). The observation that these somatic mutations are only found in a minority of cells in the tumor, combined with LOH at the POLR3A locus, strongly suggest that this subgroup of SSc represents natural cancer immunoediting (14, 15).

Like DM patients, not all SSc patients with immune responses indicative of higher cancer risk actually manifest cancer. Indeed, only approximately 20% of anti-POLR3A–positive patients have a cancer identified around the time of SSc. This suggests that either multiple mechanisms underlie the targeting of POLR3A by the immune system in SSc, and/or that cancer underlies many cases of SSc, but that in most cases the immune response is capable of controlling the cancer. In a recent study focused on SSc patients with autoantibodies against POLR3A in whom cancer does not emerge, we found that patients who also had autoantibodies against the large subunit of RNA polymerase I (RPA194) had a much lower incidence of cancer than those with antibodies against POLR3A alone (16). Because only 3 patients (3.8%) with anti-RPA194 antibodies developed cancer, it was not possible to distinguish whether this immune response identified a cancer-protective immune response versus a form of SSc unrelated to cancer.

In the present work, we studied DM patients with anti–TIF1-γ autoantibodies and examined whether patients in whom cancer did not emerge around the time of DM onset had additional autoantibody specificities compared with those in whom cancer did emerge. We selected sera with additional specificities by immunoprecipitation (IP) for further analysis, and defined 10 autoantibody specificities. We then screened for these autoantibodies in discovery and validation cohorts. Four autoantibodies were found with frequencies of greater than 6.5% in both cohorts. Cell division cycle and apoptosis regulator protein 1 (CCAR1) was the most frequent (32% in both cohorts). Anti-CCAR1 antibodies were negatively associated with cancer emergence within 3 years of DM onset. Interestingly, and distinct from our observations with anti-RPA194 antibodies in SSc, a sufficient number of cancers emerged in anti-CCAR1–positive patients over time to demonstrate that these cancers were diagnosed later after DM presentation and were more localized than those occurring in patients with anti–TIF1-γ antibodies alone. In the combined cohorts, there was also a statistically significant inverse dose-response relationship between the number of additional antibody specificities and cancer emergence. The percentage of patients in whom cancer was detected within 3 years of DM diagnosis decreased from 30% in those with autoantibodies against TIF1-γ alone, to 15%, 3%, and zero in patients with 1, 2, or more than 2 additional autoantibody specificities, respectively.

Our data demonstrate that a more diversified immune response in anti–TIF1-γ–positive DM is associated with slower emergence, or absence of, malignancy. These findings have important implications for risk stratification in DM and other autoimmune rheumatic diseases. They also provide insight into possible mechanisms underlying successful, natural regulation of human cancers by the immune system.

## Results

### More diverse autoantibody response in anti–TIF1-γ–positive DM patients that do not develop cancer.

**Figure 1:**
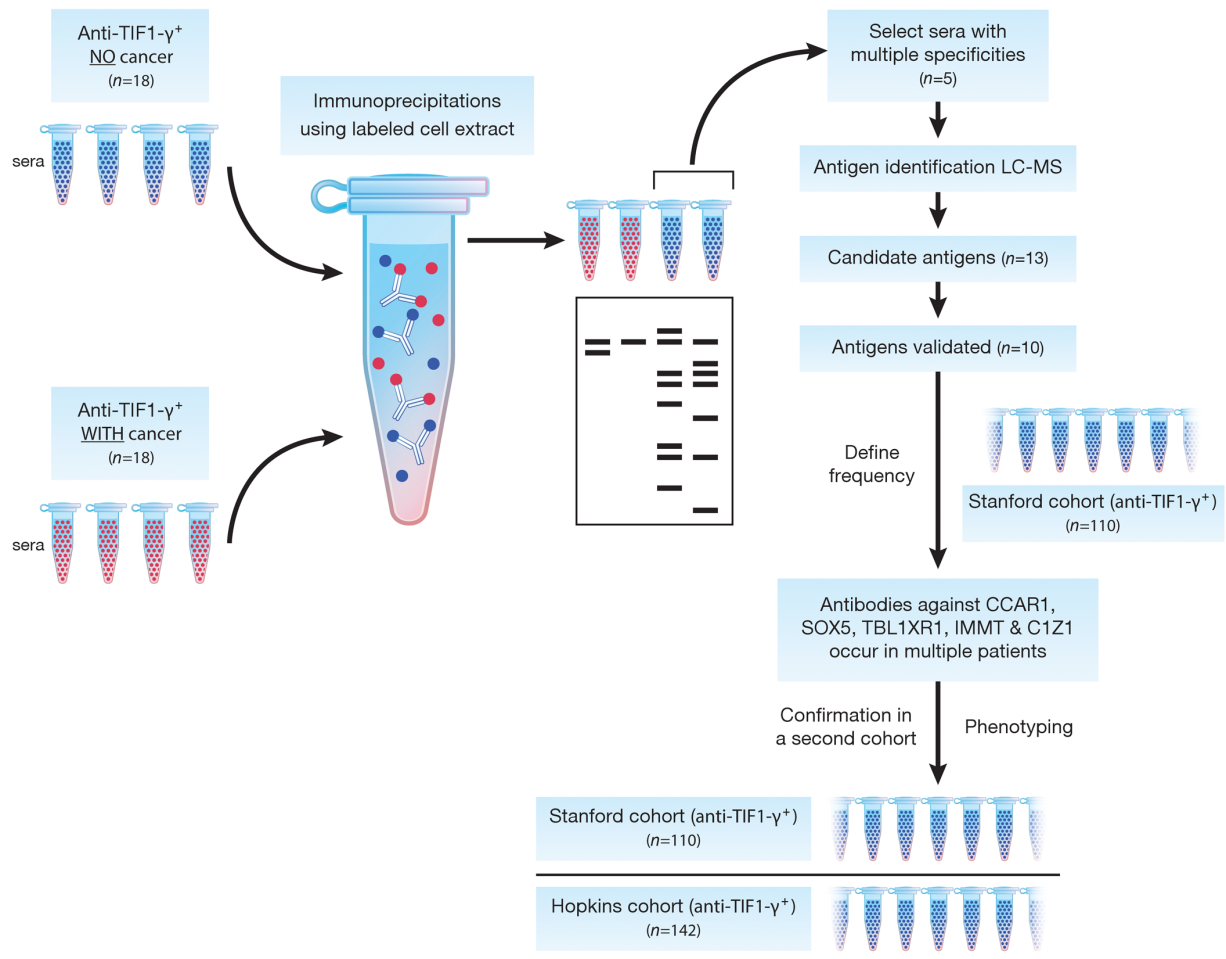
Flow diagram for autoantigen identification, validation, and phenotype association. Sera from DM patients with anti–TIF1-γ antibodies, both with (*n =* 18) and without (*n =* 18) cancer, were used to perform immunoprecipitations (IPs) with radiolabeled cancer cell line extracts as a source of antigen. Serum from 5 of the cancer-negative patients whose immunoprecipitates displayed broad antigen diversity after gel electrophoresis and subsequent visualization by autoradiography were used in larger-scale IPs. The immunoprecipitates were digested and analyzed using LC-MS. Of the identified peptides, 13 candidate antigens were selected and cognate autoantibodies were validated using recombinantly produced antigen. Validated antigens (10 total) were used to screen 110 serum samples (Stanford cohort), and those antigens targeted in multiple patients (CCAR1, SOX5, TBL1XR1, IMMT, and C1Z1) were used to screen for antibodies in a separate (Johns Hopkins) cohort for data validation. For selected analyses, data from both cohorts were combined.

**Figure 2:**
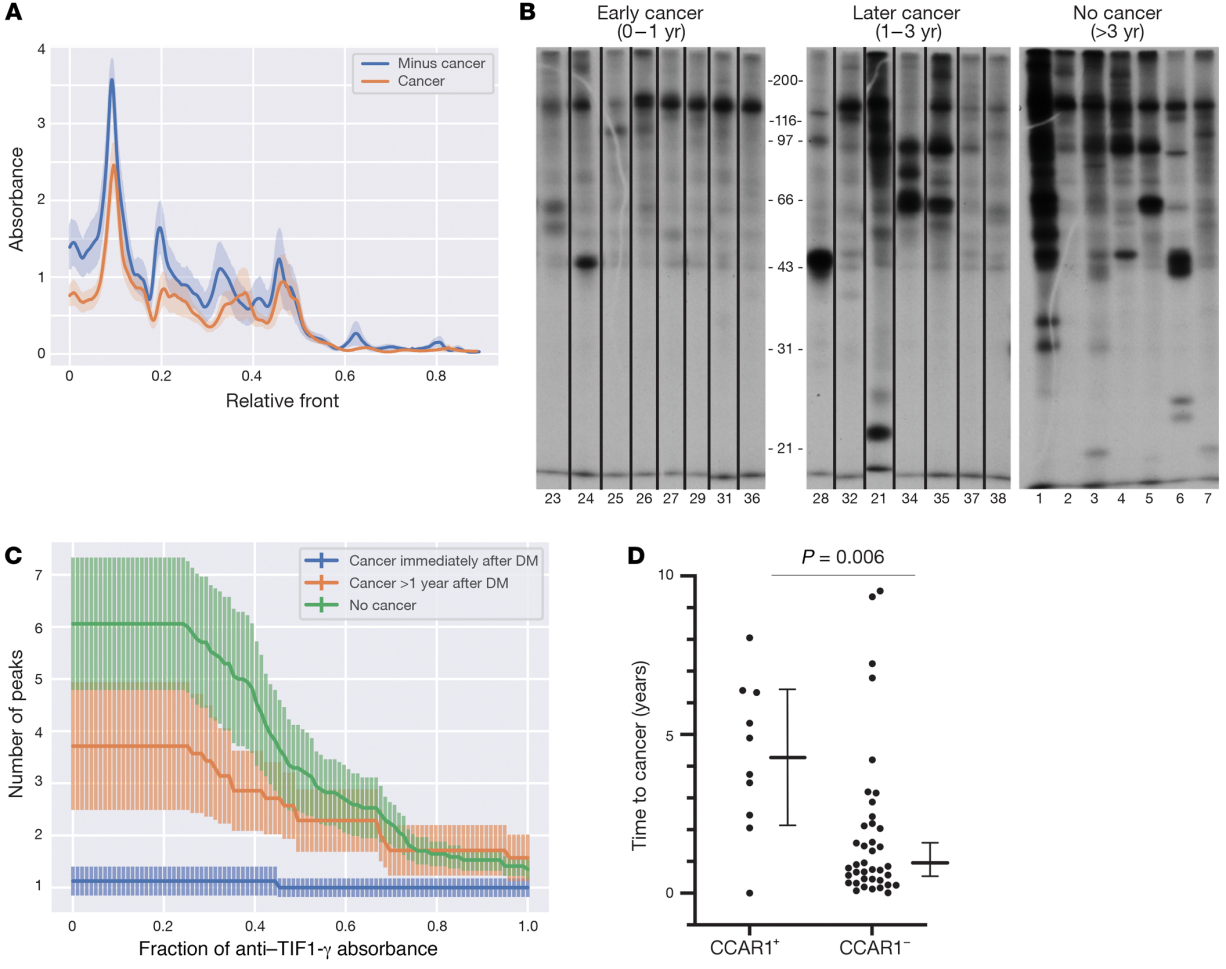
An increasing number of antibody targets is observed with lengthening time between DM diagnosis and cancer emergence. (**A**) Mean computational traces of immunoprecipitations (IPs) performed using samples from anti–TIF1-γ–positive DM patients with (*n =* 18, pink trace) or without (*n =* 18, blue trace) cancer. (**B**) IPs arranged according to cancer timing. Gel lanes from Supplemental Figure 1 were selected for presentation as follows: all samples from the left panel were selected if the cancers occurred at, or after, DM onset. Additionally, lanes 1 to 7 on the “No Cancer” gel were selected. The lanes were run in the same gel but were noncontiguous. Numbers below each lane are lane annotations used in Supplemental Figure 1. (**C**) Breadth of autoantibody targets as a function of cancer status. Antibody diversity is shown for each patient subgroup, as quantified by the number of absorbance peaks as a function of magnitude relative to the anti–TIF1-γ peak. This method captures antibody diversity that corresponds with absorbance peaks of progressively higher amplitude as *x* values increase. The response is more exclusively focused on anti–TIF1-γ in patients where cancer emerges at less than 1 year of DM diagnosis (blue), compared with the no-cancer subgroup (green), at both low and high amplitudes, which shows a strikingly broader set of autoantibody specificities. DM patients where cancer appears after 1 year have an intermediate breadth of autoantibody focus (orange). (**D**) Timing of individual cancers diagnosed after DM-symptom onset stratified by anti-CCAR1 antibody status. Distribution of delay of cancer diagnosis relative to DM onset is shown for anti–TIF1-γ–positive DM patients with (*n =* 10) and without (*n =* 39) anti-CCAR1 antibodies. All anti–TIF1-γ–positive patients (combined cohorts) with cancers diagnosed between 0 and 10 years after DM onset are shown. Median values with 95% CIs for each patient group are indicated, with *P* values for differences in medians shown (2-tailed Mann-Whitney test).

This study was designed to identify autoantibodies associated with infrequent emergence of cancer in a DM population with high cancer risk. We therefore restricted our analysis to patients with antibodies against TIF1-γ*,* since it has been shown that cancer is more likely to emerge in this DM subgroup (4). An IP approach was initially used (Figure 1). Thirty-six DM patients with anti–TIF1-γ antibodies were selected from the Stanford discovery cohort; of these, 18 had a cancer diagnosed within 3 years of DM-symptom onset, and 18 had no malignancy detected with at least 3 years of follow-up from DM-symptom onset. Plasma from these patients was used to IP proteins from radiolabeled cell lysates, and equivalently exposed fluorograms were examined to compare the IP patterns (Supplemental Figure 1; supplemental material available online with this article; https://doi.org/10.1172/JCI150201DS1). As expected, a 155 kDa band (TIF1-γ) was commonly detected as a prominent band in both sets (with and without cancer). Visual inspection of the IP data performed blinded by 3 of the investigators revealed additional autoantibody specificities in the group without cancer. This was subsequently quantitated using a signal processing analysis, which calculated mean densitometric values at any given distance of migration along the gel (described in the Methods). The resulting mean IP traces for the cancer versus noncancer groups confirmed that at several areas (corresponding to molecular weights of ~20–25 kDa, 50–60 kDa, and 85–95 kDa) the noncancer patients had a relatively higher mean density of immunoprecipitated material (Figure 2A). This result suggested that cancer-negative DM patients in general have a relatively large number of prominent autoantibody specificities.

We visualized the raw IP data (shown in Supplemental Figure 1) by digitally arranging the gel lanes in order (left to right) from short-interval cancer (<1 year), longer-interval cancer (1–3 years), and no cancer (Figure 2B). An increasing number of immunoprecipitated targets, moving from short-interval cancer to long-interval cancer to no cancer, was observed (Figure 2B). This was quantified by calculating the average number of bands relative to their intensity in each serum (described in the Methods). Using this approach, we found that, for all intensities, the short-interval cancer group had significantly fewer detectable immunoprecipitated specificities than the long-interval cancer group, which, in turn, had significantly fewer than the noncancer group (Figure 2C). We conclude that there likely exist multiple relevant specificities and that increasing numbers of immune targets are associated with decreasing chances of cancer becoming clinically apparent.

### Autoantigen discovery in anti–TIF1-γ–positive DM patients without cancer.

**Figure 3:**
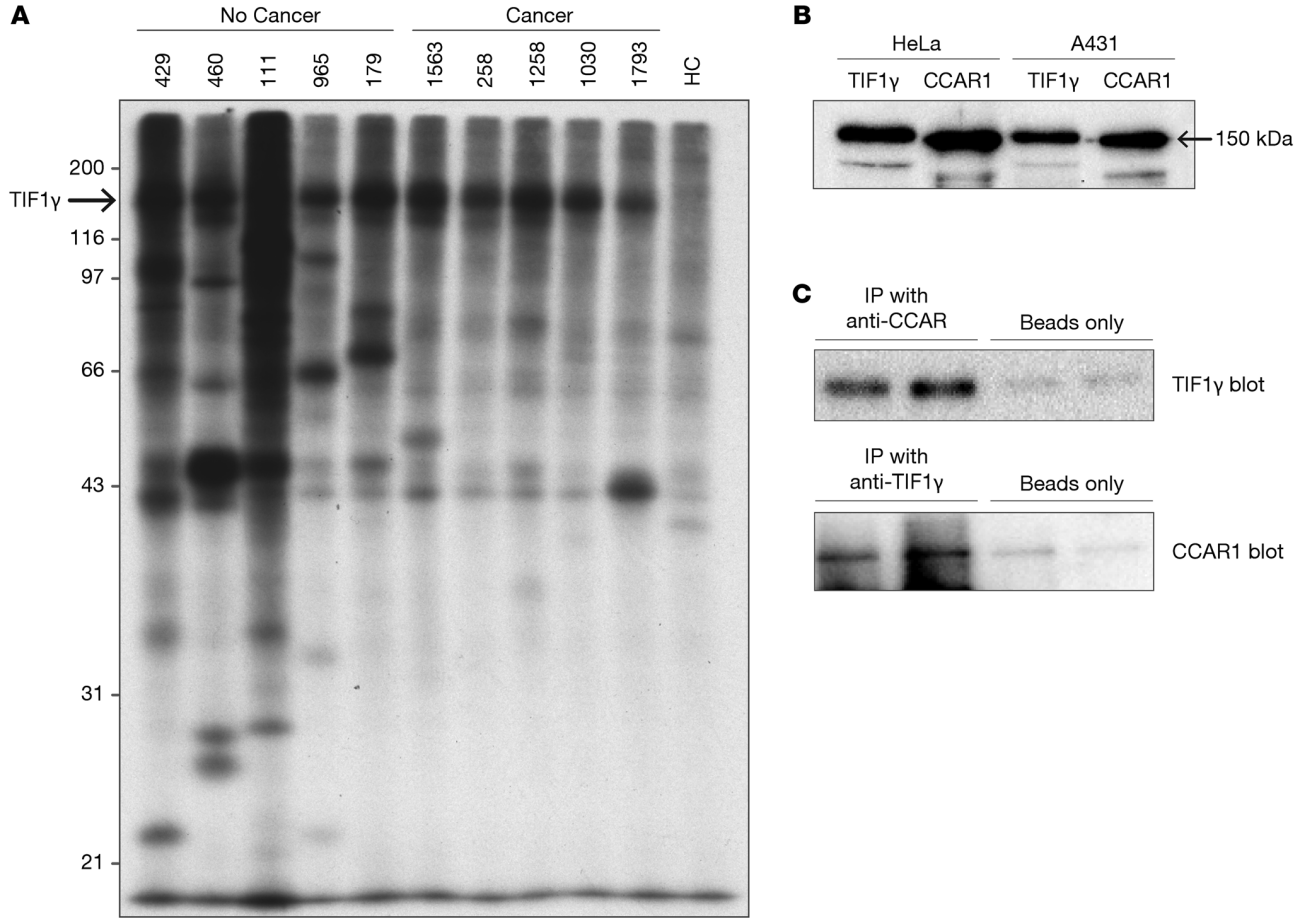
Autoantibody discovery in anti–TIF1-γ–positive DM patients without cancer. (**A**) Immunoprecipitations (IPs) were performed using lysates made from radiolabeled cells and plasma from anti–TIF1-γ–positive DM patients, 5 of whom did not have a cancer, and 5 of whom had a detected cancer. An IP performed with a sample from a healthy control (HC) individual is shown in the right-most lane. Migration of molecular weight standards is marked on the left. (**B**) Immunoblotted lysates. Lysates made from HeLa and A431 cells were immunoblotted with commercial antibodies against TIF1-γ and CCAR1, as described in the Methods section. Both proteins migrate at approximately 150 kDa. (**C**) Interaction between CCAR1 and TIF1-γ. Co-IPs were performed as described in the Methods section, using antibodies against CCAR1 (upper panel, 2 left lanes) or TIF1-γ (lower panel, 2 left lanes). Detection of the IPs was performed by immunoblotting with anti–TIF1-γ (upper panel, 2 left lanes) or anti-CCAR1 (lower panel, 2 left lanes) antibodies. Control IPs, performed using Protein A beads only, were performed and immunoblotted as above. IPs were performed in duplicate. These data are representative of those obtained in 2 additional experiments.

We therefore pursued identification of these autoantigens. For discovery, we selected (based on the most prominent IP patterns) plasma from 5 DM patients from this group of 18 for additional study. The IP profiles of the 5 selected samples are shown in Figure 3A; IPs using 5 samples from the anti–TIF1-γ–positive DM group with cancer are included for comparison. IPs from the 5 DM patients without cancer were subjected to mass spectrometry (MS) sequencing. This identified TIF1-γ in all 5 IPs (consistent with the known antibody status of these samples), as well as multiple putative autoantigens (not previously described) targeted by each sample. Of the list of 23 possible candidates that were generated, 13 were prioritized for validation based on greatest percentage coverage and availability of validation reagents (candidate hits not followed up for validation are listed in Supplemental Table 1). Validation of these putative autoantigens was performed by IP using ^35^S-methionine–labeled proteins generated by in vitro transcription and translation (IVTT) and the relevant index serum sample in each case.

Of these, 3 (ADNP, RAN, and CPSF6) were not immunoprecipitated by their index serum, while 10 autoantigens were validated (Table 1). In addition to their positive anti–TIF1-γ antibody status, 3 patients without cancer each had a single specificity identified (anti-CCAR1, -NVL2, and -NACC1), 1 patient had 2 specificities identified (anti-RCC1 and -GATD1), and 1 patient (patient 111) had 5 antibody specificities identified (anti-TBL1XR1, -KDM1A, -IMMT, -SOX5, and -C1Z1). Sera from healthy controls did not have antibodies against any of these antigens. The prevalence of each of these 10 validated specificities was determined by IVTT-IP assay in the Stanford discovery cohort, which included 110 anti–TIF1-γ–positive DM patients. Of these, antibodies against CCAR1 were by far the most frequent (detected in 35 of 110 [32%] patients; Table 2). The other specificities were found with frequencies ranging from 0.9% to 15%. Anti-CCAR1 was therefore prioritized for initial studies to investigate whether the presence of additional antibodies influenced the frequency of cancer diagnosis in anti–TIF1-γ–positive DM patients (the other specificities are addressed in the last part of the Results section). Of note, since CCAR1 and TIF1-γ have similar molecular weights and comigrate (Figure 3B), these 2 specificities cannot be accurately distinguished on the autoradiogram patterns in IPs performed on radiolabeled lysates.

### Enrichment of anti-CCAR1 autoantibodies in 2 independent cohorts of anti–TIF1-γ–positive DM patients without cancer.

We also determined the frequency of these autoantibodies in an independent validation cohort of anti–TIF1-γ–positive patients (evaluated at Johns Hopkins, *n =* 142). Demographic and clinical characteristics of patients in both cohorts are shown in Table 3. The majority of patients in both cohorts were White females. The Stanford cohort included patients who were slightly older compared with the Johns Hopkins cohort (mean age ± SD of 51 ± 16 vs. 45 ± 5). The Stanford cohort had a larger Hispanic population (21% vs. 5%). The disease duration for patients designated cancer negative was at least 3 years, but on average was greater than 9 years for both cohorts (9.7 and 9.5 years for the Stanford and Johns Hopkins cohorts, respectively). The cohorts were serologically remarkably similar, with anti-CCAR1 autoantibodies present in 32% of patients in both. They were also very similar in terms of cancer prevalence; 38 (35%) patients developed a cancer in the Stanford group, 22 (20%) of whom were within 3 years from DM-symptom onset. Similarly, a total of 44 (31%) patients developed a cancer in the Johns Hopkins cohort, 27 (19%) of whom were within 3 years of DM-symptom onset. Notably, anti-CCAR1 antibodies were very rare in patients that were negative for anti–TIF1-γ antibodies. Of 172 anti–TIF1-γ–negative patients in the Stanford DM cohort, only 1 (0.6%) tested positive for anti-CCAR1 antibodies. Interestingly, this patient also had antibodies against MDA5, NXP2, TBLX, and SOX5.

Within the Johns Hopkins cohort, there was an association between anti-CCAR1 positivity and younger age of DM-symptom onset (median 44.0 vs. 46.5, rank-sum *P =* 0.026), as well as a higher anti-CCAR1 prevalence in White patients (35% Whites positive for anti-CCAR1 vs. 7% non-White, Fischer’s exact *P =* 0.037). Similar associations were found within the Stanford cohort with regard to a younger age of DM onset (median 47.0 vs. 49.8, rank sum *P =* 0.49) and higher prevalence in Whites (35% Whites positive for anti-CCAR1 vs. 0% non-Whites, Fischer’s exact *P =* 0.009). Whereas in the Stanford cohort there was an association between female sex and anti-CCAR1–positive status (39% of women were anti-CCAR1 positive vs. 5% of men, Fischer’s exact *P =* 0.002), this was not replicated in the Johns Hopkins cohort (33% of women were anti-CCAR1 positive vs. 28% of men, Fischer’s exact *P =* 0.814). In neither cohort was an association between anti-CCAR1 and any specific cancer type present.

To address whether anti-CCAR1 antibodies are uniquely found in DM patients, or are also found in other autoimmune diseases known to have an association with malignancy, 68 sera from anti-POLR3A–positive scleroderma patients were assayed. These included 34 sera from patients with a history of cancer and 34 who had no history of cancer after at least 5 years of follow-up. Anti-CCAR1 antibodies were found in only 1 of 68 sera (1.5%) in this cohort. The anti-POLR3A–positive/anti-CCAR1–positive patient had no detected cancer, and it is noteworthy that levels of anti-CCAR1 antibodies were very low in this serum.

The relationship between anti-CCAR1 antibodies and cancer in anti–TIF1-γ–positive DM patients is shown in Table 4. In the Stanford cohort, anti-CCAR1 autoantibodies were significantly negatively associated with a diagnosis of cancer within 3 years of first DM symptom (OR 0.27 [95% CI 0.7–1.00], *P =* 0.050). Similarly, in the Johns Hopkins cohort, anti-CCAR1 autoantibodies were significantly negatively associated with a history of cancer within 3 years (OR 0.13 [95% CI 0.03–0.59], *P =* 0.008). A sensitivity analysis was performed to minimize the potential impact of immortal person-time bias, in which cancers preceding DM-symptom onset were excluded. The results were unchanged (Supplemental Table 2). In addition, the negative cancer association with anti-CCAR1 autoantibodies persisted even after controlling for potential confounders (age and biological sex) in multivariable analyses (Johns Hopkins OR 0.13 [95% CI 0.029–0.58], *P =* 0.008; Stanford OR 0.24 [95% CI 0.06–0.99], *P =* 0.049).

### A physical complex containing CCAR1 and TIF1-γ.

As noted above, antibodies against CCAR1 were restricted to the population with anti–TIF1-γ antibodies (only 1 of 172 anti–TIF1-γ–negative patients in the Stanford cohort had antibodies against CCAR1). This near-perfect association of the presence of anti-CCAR1 antibodies with concomitant antibodies against TIF1-γ suggested that an underlying mechanism driving this finding might be intermolecular epitope spreading, generally the result of association of the 2 antigens in a molecular complex (17). We therefore tested whether CCAR1 and TIF1-γ exist in a complex. IPs performed from cell lysates using polyclonal rabbit anti-CCAR1 antibodies contained TIF1-γ (Figure 3C). In a reciprocal strategy, CCAR1 was found to be present in IPs done from cell lysates using a rabbit monoclonal anti–TIF1-γ antibody (Figure 3C), together demonstrating that these molecules are found in the same complex.

### Later appearance and less advanced stage in cancers from anti-CCAR1–positive patients.

We observed that cancers were sometimes diagnosed in anti-CCAR1–positive patients (10 in the Johns Hopkins cohort, 8 in the Stanford cohort), and wondered whether these cancers have similar timing of diagnosis and cancer stage as those cancers diagnosed in anti–TIF1-γ–only DM patients. To investigate this, we recorded the time of cancer appearance (relative to DM onset) and the cancer stage of all 82 patients with malignancies in this study (see Supplemental Table 3 for the complete list of cancer types, timing, and stage). The similarity in clinical evaluation, data collection, and consistency of the anti-CCAR1–positive associations in the 2 cohorts allowed us to pool them. The increased statistical power afforded by this enabled us to address whether the stage of the cancer or time to cancer diagnosis after DM onset differed in anti-CCAR1–positive versus anti-CCAR–negative patients. To facilitate interpretation, we excluded cases in which cancer preceded DM onset, or emerged more than 10 years after DM onset.

A total of 10 anti-CCAR1–positive patients with cancer met these criteria, 9 of whom had staging data (Table 5). Of these 9 patients, 8 (89%) were diagnosed at low stage (0 or 1) and only 1 patient (11%) had a stage of 2 or greater. In contrast, patients with anti–TIF1-γ autoantibodies alone had significantly fewer cancers at low stage (14 of 33 [42%], *P =* 0.02). We considered the possibility that our data pertaining to stage could be a result of anti-CCAR1–associated cancers being enriched for types that typically present at an earlier stage. While cancer types were largely similar in the anti-CCAR1–positive versus anti-CCAR1–negative autoantibody groups, the anti-CCAR1–negative group had 3 ovarian cancers, whereas the anti-CCAR1–positive group had none. Because ovarian cancer uniquely presents at a more advanced stage relative to other cancers (18), we ran a sensitivity analysis that excluded the cases of ovarian cancer; enrichment of low-stage cancers in the anti-CCAR1–positive group remained significant (*P =* 0.05, Fischer’s exact test).

We next looked at the time interval between DM-symptom onset and cancer appearance (Figure 2D). Patients positive for anti-CCAR1 antibodies were diagnosed with cancer significantly later compared with anti-CCAR1–negative patients (median time from DM onset 4.3 vs. 0.85 years, respectively; *P =* 0.006). Of note, this cannot be explained by differences in follow-up time, as this was similar in anti-CCAR1–positive and anti-CCAR1–negative cancer-free patients in both cohorts (Johns Hopkins cohort, median follow-up 8 years in anti-CCAR1–negative patients, 10 years in anti-CCAR1–positive; Stanford cohort, median follow-up 9 years in anti-CCAR1–negative patients, 10 years in anti-CCAR1–positive).

For both the stage and time analyses, sensitivity analyses were performed to include cancers occurring 6 months prior to DM-symptom onset. The rationale for this inclusion is that there is inherent imprecision in estimating the date of DM onset. Thus, cancers occurring within 6 months of DM-symptom onset were considered to be contemporaneous in this sensitivity analysis. An additional 5 anti-CCAR1–negative and no anti-CCAR1–positive patients were included in “time zero.” Of these 5 anti-CCAR1–negative patients, 4 (80%) had a cancer stage of 2, 3, or 4. Our results were strengthened in this analysis for comparison of stage (89% vs. 39%, *P =* 0.01 by Fischer’s exact test). Similarly, for the time analysis in anti-CCAR1–positive vs. –negative patients, the median time of cancer diagnosis from DM onset was 4.3 vs. 0.74 years, respectively; *P =* 0.002).

### Additional antibodies and their relationship with cancer.

The finding that anti-CCAR1 antibodies are enriched in patients in whom a cancer never emerges, or emerges after a time delay, prompted us to test whether this observation holds for the other autoantibody specificities identified within the anti–TIF1-γ–positive population (Table 2). Of the 9 additional new specificities, 3 were found in the index case alone (anti-GATD1, -RCC1, and -KDM1A), while 1 was found only in the index case plus an additional patient (anti-NVL2). The remaining 5 were detected in multiple patients, with frequencies ranging from approximately 2.5% to 21% of the anti–TIF1-γ–positive patients (anti-NACC1, -C1Z1, -IMMT, -TBL1XR1, and -SOX5).

Comparing the 2 cohorts, a striking similarity in the prevalence and rank order of the 10 specificities was observed (Table 2). In both cohorts, approximately half of the patients produced autoantibodies in addition to those against TIF1-γ; 30% produced 1, and approximately 20% produced 2 or more. Upon dichotomizing the 10 autoantibodies to zero (anti–TIF1-γ only) versus any (anti–TIF1-γ “plus”), large differences in cancer frequency were observed. In the Johns Hopkins cohort, there was a 4-fold higher frequency of cancer in the anti–TIF1-γ–only group compared with patients who produced any of the 10 autoantibodies; cancer emerged in 37% of patients with anti–TIF1-γ only versus 9% with anti–TIF1-γ “plus” within 5 years, 34% versus 7% within 3 years, and 27% versus 4% within 1 year. In the Stanford cohort, the cancer frequency was 2-fold higher; cancer emerged in 32% versus 19% within 5 years, 27% versus 13% within 3 years, and 20% versus 4% within 1 year.

Combining both cohorts, we analyzed the number of autoantibody specificities patients produced in relationship to cancer diagnosis (Table 6). For all DM-onset/cancer time intervals, a dose-response relationship was observed; as the number of autoantibody specificities patients produced increased, the frequency of cancer decreased. These trends were most notable for cancer within 3 and 1 year (Table 6; Fisher’s exact *P <* 0.001 for all trends).

**Figure 4:**
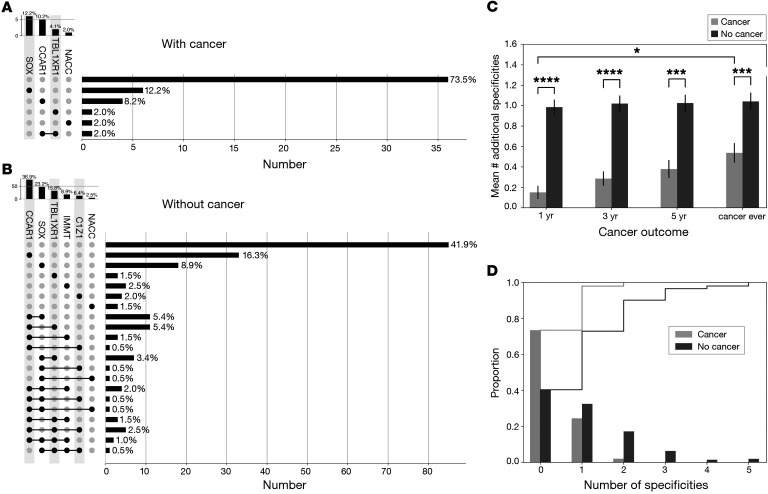
Protection against cancer is associated with combinatorial expression of autoantibodies. (**A** and **B**) Number, identity, and frequency of unique autoantibody combinations in patients with (**A**) or without (**B**) cancer within 3 years of DM onset. The vertical histogram above the matrix shows the frequency of specific autoantibodies in the cohort of anti–TIF1-γ–positive patients, in order of decreasing magnitude. The *y* axis of those plots denotes number of patients. In the matrix itself, each row represents 1 autoantibody combination. Gray circles denote absence of a specific antibody, black circles denote presence, and when multiple specificities are present in a combination, they are connected by black lines. The frequency of each combination is shown in the horizontal bar plots; the *x* axis denotes the number of patients. (**C**) Mean number of autoantibody specificities in anti–TIF1-γ–positive DM patients in whom cancer does or does not emerge. Data at 1 year, 3 years, 5 years, and ever after DM diagnosis are shown (mean ± SEM, obtained by a bootstrapping procedure [*n =* 10,000 samples]). *P < 0.05; ***P < 0.001; ****P < 0.0001 by 2-tailed, independent 2-sample *t* test. (**D**) Proportion of DM patients in whom cancer does (“cancer”) or does not (“no cancer”) emerge within 3 years of DM diagnosis that have anti–TIF1-γ antibodies only (0) or anti–TIF1-γ plus additional specificities (1–5). Histograms of antibody count in excess of anti–TIF1-γ were computed and are shown. The cumulative distribution of antibody count in excess of anti–TIF1-γ was also computed for cancer versus no-cancer groups, and superimposed on the histograms (thin traces at top).

To further understand the relationship between combinations of autoantibodies and cancer emergence, we visualized the distribution of all such combinations in patients with versus without cancer at different intervals around DM onset using UpSet, a novel visualization tool for the quantitative analysis of overlapping subsets (Figure 4, Supplemental Figure 3, and ref. 19). We reasoned that novel insights might be gained using a combinatorial analysis rather than focusing on a single autoantibody at a time. Several interesting features were evident: (a) in patients with cancer ±3 years, 74% had anti–TIF1-γ antibodies alone. The remaining 26% either had anti-CCAR1 or anti-SOX5 in isolation, and only 1 of these patients (2% of the group with cancer) had them in combination with another antibody (Figure 4A). In contrast, only 42% of those without cancer at 3 years had anti–TIF1-γ antibodies alone (Figure 4B). Some patients in this group had single additional antibodies from the group of 10 novel autoantibodies, most frequently against CCAR1 (16%) or SOX5 (9%). Particularly striking were the 14 combinations of multiple autoantibodies (Figure 4B). These were present in 25.4% of patients and always involved combinations including anti-CCAR1 (11.4%), anti-SOX5 (4.8%), or both (9.2%). (b) While anti-CCAR1 antibodies in isolation were enriched in patients without cancer, anti-SOX5 autoantibodies in isolation did not have a similar association. (c) The mean number of additional autoantibody specificities in DM patients with cancer was 0.15 in patients with cancer ±1 year, rising to 0.38 in patients with cancer ±5 years. In contrast, the mean number of additional specificities for patients without cancer was 1 at all time points. The difference was statistically significant at all time points (Figure 4C). (d) When anti-CCAR1 antibodies were present, they occurred alone in 46% (37 of 80) of patients, and in combination in 54% (43 of 80). Similarly, isolated anti-SOX5 antibodies occurred in 45% (24 of 53) of anti-SOX5–positive patients, and in combination in 55% (29 of 53). Anti-TBL1XR1 antibodies were strikingly different; they were present in isolation in only 11.7% (4 of 34) of patients, and were found in combinations with other specificities in 88.3% (30 of 34) (distributions between solo and combination were strikingly different for anti-TBL1XR1 and anti-CCAR1 [*P <* 0.0005], as well as anti-TBL1XR1 and -SOX5 [*P <* 0.001]). Anti-TBL1XR1 autoantibodies therefore appear to arise mainly in the setting of immune responses against CCAR1, SOX5, or both.

## Discussion

Despite evidence that somatic mutation in cancer can trigger autoimmunity to a specific molecular target in rheumatic diseases (14, 20), several observations are not explained by this simple concept: first, even in high-risk cancer antibody subgroups, the majority of patients never develop a cancer (4), and secondly, among patients who do develop a cancer, the timing of cancer emergence is heterogeneous (6, 21, 22). A key question is whether this heterogeneity represents a stochastic process, or is mechanism based, reflecting a spectrum of immune responses with increasing anticancer efficacy. Since many of the clinically relevant DM phenotypes and trajectories are marked by distinct autoantibodies (23), we wondered whether there are additional autoantibody specificities within the anti–TIF1-γ autoantibody–positive subset that might explain those patients in whom cancer either does not emerge, or emerges late.

In a discovery cohort (Stanford) of well-phenotyped anti–TIF1-γ–positive DM patients, we initially demonstrated that those without cancer had additional specificities compared with those with cancer. This prompted us to define and identify additional autoantigens in anti–TIF1-γ–positive patients without cancer. There are many approaches to defining previously undiscovered autoantibodies. Based on our prior experience (13) showing that the focus of many human autoantibodies is overwhelmingly on conformational and discontinuous epitopes, we selected an approach coupling IP using patient immunoglobulins with on-bead digestion and MS-MS sequencing for autoantigen identification.

We identified and validated 10 additional autoantibodies in these anti–TIF1-γ–positive patients. They fell into 2 categories: those more frequently targeted across DM patients, and those present almost exclusively in single patients from the initial screening cohort. Autoantibodies against CCAR1 were most frequent in anti–TIF1-γ–positive DM sera (found in 32% in both cohorts studied) and were associated with striking decreases in the odds of cancer occurring within 3 years in patients with these antibodies (OR 0.13–0.27), strongly suggesting that the combined immune response marks a subgroup in which cancer is much less likely to emerge. Interestingly, rare cancers nevertheless did occur in the anti-CCAR1–positive group, giving us the opportunity to ask whether there was anything distinct about cancers emerging in the setting of the combined immune response. Although the numbers were small, these data showed that cancers were diagnosed later in anti-CCAR1–positive patients than their anti-CCAR1–negative counterparts (median time from DM onset 4.3 vs. 0.85 years, respectively; *P =* 0.006).

The relatively tight clustering of cancer presentation around the time of DM diagnosis in patients whose immune response remains focused on TIF1-γ alone is striking. Furthermore, 89% of anti–TIF1-γ–positive patients with anti-CCAR1 antibodies were diagnosed at stage 0 or 1, which contrasts with 42% of anti–TIF1-γ–positive DM patients without anti-CCAR1 antibodies, who largely presented with more advanced cancers. Interestingly, there are other anti–TIF1-γ–positive/anti-CCAR1–negative DM patients who behave similarly to anti-CCAR1–positive patients (that is, no cancer, or delayed cancer emergence); the size of this population is at least equal to the anti-CCAR1–positive group (Figure 2D). Serum from these patients was not positive for the autoantibodies defined in these studies, strongly indicating that additional specificities that predict slow or absent cancer emergence remain to be discovered. Additional studies to define these specificities are underway. It is possible that use of additional cell lines derived from a range of cancers relevant to the DM disease spectrum as antigen sources may be helpful to discover additional novel autoantigens in this anti-CCAR1 antibody–negative group.

Interestingly, CCAR1 autoantibodies in DM were restricted to the anti–TIF1-γ–positive group and were not found in DM patients with other autoantibody specificities. This striking linkage of the 2 immune responses is likely mechanistically driven. A known characteristic of the autoantibody response is its ability to spread to multiple components of multimolecular complexes by intermolecular spreading (17). We confirmed that TIF1-γ and CCAR1 could be coprecipitated from cells (Figure 3C). It is possible that the constituents and behavior of this complex varies in different cancers and their microenvironments, and that such differences influence the initiation and propagation of the immune response to the components of the complex in some DM patients. It is also possible that anti-CCAR1 autoantibodies might be inhibiting a cancer-promoting function of anti–TIF1-γ.

In addition to anti-CCAR1, we identified another 5 autoantibodies found in anti–TIF1-γ–positive DM patients at frequencies varying from 2.5% to 21%, with the second most frequent antibody targeting SOX5. We used UpSet plots to analyze the association of autoantibody combinations with the emergence of cancer in anti–TIF1-γ–positive DM patients. Combinations of multiple autoantibodies were present in approximately 25% of DM patients without cancer, but were very rare in patients in whom cancer emerged within 3 years (2%), or in the first year (0%). Despite the fact that these antigen specificities were independently identified, 4 of the 10 (CCAR1, TBL1XR1, IMMT, and CIZ1) defined a cluster in which members were frequently found in combination. It is of interest that TBL1XR1, IMMT, CIZ1, and SOX5 were all identified initially from a single patient. These specificities were subsequently found to be frequent in DM patients without cancer, where they occurred in various combinations. Since 42% of anti–TIF1-γ–positive patients without cancer lack the 10 autoantibodies defined here, it is likely that there are still prominent autoantigens that await definition in the no-cancer group, and that these will not overlap with the cassette of autoantigens defined in these studies. It is presently unknown how heterogeneous such a group may be, and whether distinct functional patterns associated with cancer attenuation may become evident. Additional studies to define these specificities are underway.

**Figure 5:**
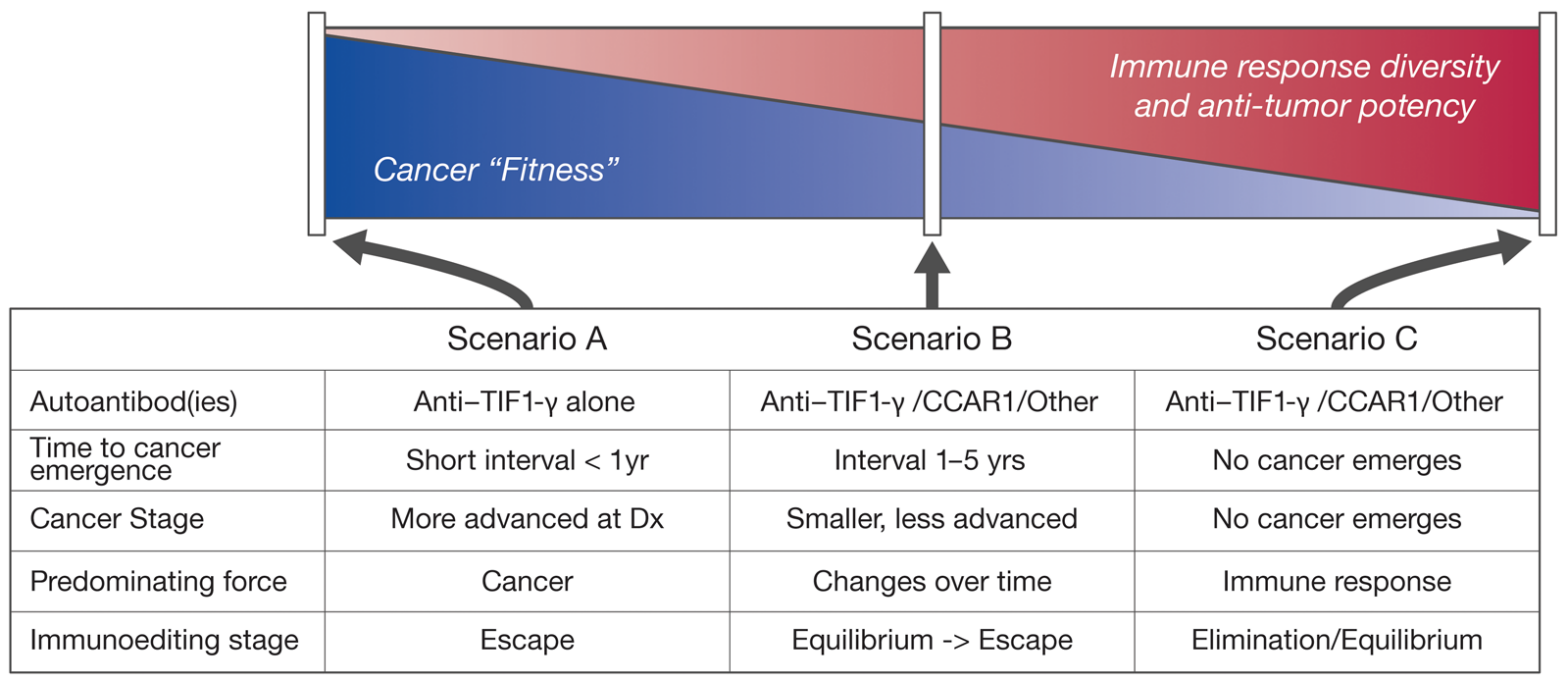
Model of relationship between cancer fitness and immune response. Model is depicted as a spectrum of decreasing cancer fitness (left to right) and its inverse relationship with the antitumor immune response. All scenarios represent DM in association with anti–TIF1-γ antibodies and incipient cancer. Scenario A is a state of high cancer fitness with a paucity of additional immune responses beyond anti–TIF1-γ. This part of the spectrum is associated with rapid (around time of DM onset) and aggressive (e.g., advanced stage) cancer emergence (“immune escape”). Scenario B represents a balance between cancer and immune response (equilibrium), and is characterized by a broader immune response (e.g., anti-CCAR1). In this scenario, cancer eventually manifests (a transition from equilibrium to immune escape), but is less aggressive (e.g., earlier stage) and emerges after a time delay following DM onset. Scenario C is also characterized by a broad (e.g., anti-CCAR1) and effective immune response, but is one in which the antitumor response ultimately deletes (elimination) or maintains the cancer in a subclinical state (equilibrium). Note that other mechanisms exist that might explain the relationship of additional autoantibodies with attenuated cancer emergence (e.g., additional autoantibodies attenuate a procancer property of anti–TIF1-γ).

Our data suggest that the cancer-DM relationship is a continuum that can be explained within the framework of cancer immunoediting, with important insights provided by the human disease model. Cancer immunoediting is thought to have 3 functional phases (not necessarily separable, or of a particular duration) — elimination, equilibrium, and escape (24). It has been proposed that during development of a cancer, the mutanome provides a spectrum of neoantigens against which the host immune system responds (25). When particular immune responses target autoantigens associated with autoimmune damage of specific tissues (e.g., TIF1-γ), we propose that specific autoimmune phenotypes (e.g., DM) emerge (15, 26). The data in this study are consistent with a model in which the breadth of the immune response influences whether cancer will emerge (escape phase) or remain silent (elimination or equilibrium phases). The striking temporal clustering of DM and cancer diagnosis when patients make an isolated immune response against only TIF1-γ suggests that the breadth of the immune response influences the pace of movement through the phases of immunoediting to escape (Figure 5, scenario A). The later emergence of cancers in patients in which the immune response has targeted multiple autoantigens likely reflects cancers whose growth has been restrained (i.e., cancer equilibrium and then escape; Figure 5, scenario B), to cancers whose emergence is prevented (i.e., cancer elimination or equilibrium; Figure 5, scenario C). Although we have used autoantibodies to discover target antigens, it is likely that multiple immune effector pathways (particularly cellular cytotoxic pathways mediated by CD8^+^, CD4^+^, and NK cells) are most relevant to any anticancer effect of these immune responses. While there have been noteworthy descriptions of in vitro and in vivo anticancer effects of anti-DNA antibodies, these required specific susceptibilities in the cancer to observe the effects (in that case, defects in DNA repair pathways; refs. 27, 28). Additional studies to define the mechanisms whereby specific immune responses exert anticancer effects are a high priority. Defining whether the other molecules besides CCAR1 also associate physically with TIF1-γ will provide additional insights into the potential mechanisms underlying the targeting of this autoantigen cluster.

The decreased frequency of cancer observed in anti-CCAR1–positive patients was present across multiple cancer types, suggesting that the effect is not limited to a particular tumor type or mechanism. Interestingly, chemical inhibitors of CCAR1 function have been shown to negatively affect the viability of multiple types of cancer (29, 30). In this context, we propose that immunization with linked sets of antigens (e.g., TIF1-γ, CCAR-1, or the other autoantigens) associated with cancer protection as defined in this study might be harnessed in novel prevention and therapeutic approaches to cancer, particularly in high-risk groups.

The finding of multiple specificities of broad frequency in anti–TIF1-γ–positive patients who remain cancer free or where cancer is delayed is of interest. The mechanisms underlying the additional immune responses in anti–TIF1-γ–positive DM patients remain unclear. For some frequently targeted autoantigens (e.g., SOX-5, mutated in 8.6% of cancers in the Catalogue Of Somatic Mutations In Cancer [COSMIC]; https://cancer.sanger.ac.uk/cosmic), it is possible that the immune responses target somatic mutations in incipient cancers. For anti-CCAR1, which is also frequently targeted but is less frequently mutated in cancer (2.1% of cancers in COSMIC), the observations here that CCAR1 and TIF1-γ are in a molecular complex strongly indicate that CCAR1 is targeted through intermolecular spreading. Many of the remaining infrequently targeted antigens are also infrequently somatically mutated in cancers and may represent immune responses to the cancer mutanome. While these questions cannot be addressed in scleroderma, or in DM where cancer never emerges, the small group of DM patients with cancers that emerge late may provide an important opportunity to further examine these mechanisms.

The incidence of cancer has long been recognized to be increased in patients with DM, and to cluster in the several-year period around the appearance of DM (1, 31). It is noteworthy, however, that cancer is only diagnosed in a minority of DM patients. A major gap in the management of patients with DM therefore remains lack of information and tools to identify those patients at the highest risk of developing a cancer. As a consequence, cancer screening is broadly applied. The identification of anti–TIF1-γ as a marker of a subgroup at high risk of malignancy (32, 33) was of clinical interest in this regard, but the majority of patients with these autoantibodies still fail to develop cancer. Our new findings, made in 2 separate patient cohorts, that additional autoantibody specificities in anti–TIF1-γ–positive patients are associated with a low incidence of cancer (anti-CCAR1, -TBLX1R1, -IMMT, and -CIZ1), will find rapid application in improved risk stratification to guide clinical evaluations in newly diagnosed DM. In the current study, plasma from 5 DM patients with no detected malignancies were used for novel autoantigen discovery. It is likely that screening additional samples from patients negative for these autoantibodies and additional antigen sources will yield more new specificities after processing through this discovery pipeline. Expanding this approach in DM and other rheumatic diseases with a cancer association will likely further improve the prediction of imminent cancer.

This work provides additional support for the utility of the rheumatic diseases as powerful models to define the role of autoimmunity in immune regulation of human cancer. Learning the features of the effective natural anticancer immune response in these diseases may find additional applications in enhancing cancer immunotherapies.

## Methods

### Study design.

The objective of this study was to identify autoantigens preferentially targeted by patients in whom cancer did not emerge, utilizing a high-cancer-risk DM population (defined as having anti–TIF1-γ antibodies). This was a retrospective cohort design in which the study population consisted of DM patients seen in the outpatient clinics at Stanford and Johns Hopkins (see start and end dates below) who consented to donate blood, and who were found to have anti–TIF1-γ autoantibodies in their serum. Sera from 172 DM patients without anti–TIF1-γ antibodies who were part of the same population seen at Stanford were used as control samples to assess phenotype restriction of novel antibodies.

### Stanford DM cohort (discovery cohort).

All patients were seen in the outpatient clinics of the Stanford University Department of Dermatology between July 2004 and August 2017. Of the 110 patients, 92% met probable or definite by 2017 ACR/EULAR IIM classification criteria (34). All clinically amyopathic patients met Sontheimer’s criteria for this phenotype (35). All patients had onset of DM after 18 years of age. Clinical data were abstracted from the study database. DM onset was defined as either the date of first rash or muscle weakness, whichever came first.

### Johns Hopkins DM cohort (validation cohort).

All patients were seen in the outpatient clinics of The Johns Hopkins Myositis Center between January 2007 and December 2017. All participants in the study met the definition of probable or definite DM by Bohan and Peter criteria (36), and 141 of 142 (99%) met probable or definite DM by 2017 ACR/EULAR IIM classification criteria. To capture patients with clinically amyopathic DM, patients with Gottron’s and/or heliotrope sign with interface dermatitis on skin biopsy were also included. All patients had onset of DM after 18 years of age. Clinical data were abstracted from the Hopkins Myositis database and from the electronic medical record. DM onset was defined as first symptom as reported by patient including rash, weakness, myalgia, or dyspnea.

### Healthy controls.

Serum was obtained from 34 healthy control subjects. The Johns Hopkins IRB approved the protocol, and all individuals provided written informed consent.

### Scleroderma cohort.

The scleroderma cohort consisted of sera from 68 well-characterized scleroderma patients with anti-POL3A antibodies evaluated at the Johns Hopkins Scleroderma Center. Thirty-four sera were from patients with a history of cancer, and 34 were from patients who had no history of cancer after at least 5 years of follow-up. These sera were all part of the cohort used and are fully described in Shah et al. (16). The Johns Hopkins IRB approved the protocol, and all individuals provided written informed consent.

### Cancer screening and definitions.

Timing and methodology for cancer screening was determined by the treating physician. The vast majority of patients received CT scanning of chest, abdomen, and pelvis at least once during the first 3 years following DM onset, in addition to age- and sex-appropriate cancer screening. In the Hopkins cohort, of the 142 patients studied, 79% received at least 1 CT chest and 71% received at least 1 CT abdomen/pelvis scan for cancer surveillance, in addition to age- and sex-appropriate screening. In the Stanford cohort, of the 110 patients studied, 81% received at least 1 CT chest and 84% received at least 1 CT abdomen/pelvis scan for cancer surveillance. Cancer was defined as any malignancy diagnosed with tissue biopsy, excluding nonmelanoma cancer of the skin. The American Joint Committee on Cancer (AJCC) staging classification system was used to define stage of cancer at the cancer index date. Cancer index date was defined as the date of cancer diagnosis, or, in cases where the cancer was in clinical remission and later recurred, either the date of recurrence or original diagnosis was used, whichever was closest to date of DM-symptom onset.

### Cell cultures and immunoblotting.

HeLa, A431 (both purchased from ATCC), and Mel 624 melanoma cells (gift from Suzanne Topalian, Johns Hopkins University) were cultured using standard tissue culture procedures. For the immunoblots shown in Figure 3B, A431 and HeLa cells were washed extensively with PBS before lysing with buffer A (1% Nonidet P-40, 20 mM Tris [pH 7.4], 150 mM NaCl, 1 mM EDTA, and a protease inhibitor cocktail). Cell lysates (20 μg/lane for the TIF1-γ blots and 5 μg/lane for the CCAR1 blots) were electrophoresed in 10% SDS-PAGE gels and transferred to nitrocellulose membranes. Immunoblots were performed using a rabbit polyclonal anti-CCAR1 antibody (Novus Biologicals, NB500-186; 1:7,500 dilution) or a mouse monoclonal anti–TIF1-γ antibody (Novus Biologicals, H00051592, clone 6D1; 1:1,000 dilution), followed by incubation with horseradish peroxidase–labeled secondary antibodies (Pierce) and chemiluminescence. Images were acquired using a Protein Simple Fluorochem-M digital imager. The same antibodies were used for blotting the immunoprecipitations shown in Figure 3C and Supplemental Figure 2.

### Sample collection and anti–TIF1-γ antibody ELISA.

Plasma/serum was obtained from all DM patients from both cohorts on (or within 6 months) the date of their initial clinic visit and aliquots were banked at –80°C. The same sample was used for all autoantibody testing in the study. Antibodies against TIF1-γ were determined by ELISA using a commercially available ELISA kit (MBL) as previously described (37). The cutoff for antibody positivity was set at 7 units; this value was based on the mean + 4 SD of values obtained from 67 healthy controls banked at the Johns Hopkins site that were assayed with this kit. Of note, a comparison of the anti–TIF1-γ antibody status obtained using this ELISA compared to those obtained with an IP/immunoblot (IP/blot) assay (described in refs. 9, 37) gave similar results overall, with the ELISA being more sensitive (able to detect anti–TIF1-γ antibodies at 7 units, while the lower limit for detection with IP/blot was in the 10–15 unit range (Supplemental Figure 2A).

### IP using ^35^S-methionine–labeled IVTT proteins to detect antibodies.

Complementary DNAs (cDNAs) encoding full-length FLAG-tagged human CCAR1, RCC1, GATD1, TBL1XR1, KDM1A, IMMT, SOX5, C1Z1, NVL2, and NACC1 were purchased from GenScript. All DNAs were sequence verified before use. ^35^S-methionine–labeled proteins were generated from these cDNAs by IVTT reactions, per the manufacturer’s protocol (Promega). IPs performed using these products as input material were electrophoresed in 10% SDS-PAGE gels and visualized by fluorography as described previously (38). IPs performed with a positive reference serum (anti-SOX5 and anti-C1Z1) or an anti-FLAG IP (all other IVTT products; antibody from Sigma-Aldrich, F1804, clone M2) were included in each sample set, and fluorogram exposures were standardized to give reference IP bands a similar intensity. Positive/negative antibody status was assigned by independent visual inspection of the equivalently exposed autorads by 2 skilled investigators. All samples assigned a positive antibody status (and a subset of the negative samples) were assayed a second time to confirm positivity. Sera from healthy controls banked at the Johns Hopkins site were also tested by IP with each of the IVTT products. No IP band was detected with any of the control sera (see Supplemental Figure 2B for representative examples).

### IPs performed from radiolabeled Mel 624 cell lysates.

Mel 624 cells were radiolabeled with ^35^S-methionine and used for IPs performed with patient plasma as described previously (16). The IPs were electrophoresed in 10% SDS-PAGE gels and visualized by fluorography. An IP performed with the same anti-PMSCL reference serum was included in each set to standardize exposure intensities (Supplemental Figure 1, lanes 20 and 39).

### Computational analysis of IP traces.

Fluorograms of electrophoresed IPs (equivalently exposed based on the intensity of the anti-PMSCL reference IP bands) were scanned by densitometry (Bio-Rad software), producing a vector of absorbance values for each patient, with higher numbers corresponding to darker fluorogram bands. Comparability of absorbance values across patients was ensured by first smoothing with a Gaussian filter of width 1 (to account for noise in the densitometer’s reads), and aligning to the TIF1-γ peak (the first and highest peak on each trace, at ~0.1 relative front). The mean across patients in each group was then computed separately, and the SEM at each point (shown as CIs) was computed using a bootstrapping procedure. Bands were distinguished by applying a standard peak-finding procedure (implemented in Scipy’s signal package) to the smoothed vector of absorbance values. In each serum, peaks were sorted by their amplitude (absorbance) and expressed as a fraction of that serum’s TIF1-γ absorbance. These values were used to compute the mean number of bands that are between 0 and 100% as high as the TIF1-γ peak for each disease subgroup, as well as the standard error of the subgroup sample mean at each point.

### Identification of new antibody specificities by MS.

IPs were performed as described above, using lysates made from unlabeled Mel 624 cells and selected plasma samples from patients without cancer. The amount of lysate and plasma used per IP for these assays was scaled up 5-fold relative to the radiolabeled IPs. Further processing was performed at the Johns Hopkins University Proteomics Core facility as follows. Briefly, on-bead digests were performed with trypsin/LysC and the resulting peptides were analyzed by reverse-phase LC-MS. Eluting peptides were sprayed into a Q-Exactive Plus (QE Plus, Thermo Fisher Scientific) mass spectrometer. Isotopically resolved masses were extracted using Proteome Discoverer software and searched using Mascot 2.5.1 through Proteome Discoverer against a human protein database. Peptide identifications from Mascot searches were processed within Scaffold (Proteome Software) with display criteria set to 95% confidence for both protein and peptide identifications.

### Co-IP of TIF1-γ and CCAR1.

HSG cells (gift from Bruce Baum, NIH) were treated with 50 mM etoposide (Cell Signaling Technology) for 3 hours, and then washed with PBS on ice and lysed in RIPA buffer (50 mM Tris pH 7.4, 150 mM NaCl, 5 mM EDTA, 0.5% Nonidet P-40, 0.5% sodium deoxycholate, 0.1% SDS, with phosphatase inhibitors and protease inhibitors added). After centrifugation at 16,000*g* (20 minutes, 4°C), the supernatants were diluted in buffer A, precleared with Protein A beads, and then used as input for IPs. These were performed by incubating with (a) an anti-CCAR1 rabbit polyclonal antibody (Novus Biologicals, NB-500-186) or (b) an anti–TIF1-γ rabbit monoclonal antibody (Cell Signaling Technology, 90051, clone D7U4F) for 90 minutes at 4°C, followed by addition of Protein A–agarose beads (25 minutes, 4°C). Control IPs were performed by omitting the primary antibody, and incubating with Protein A beads only. After extensive gentle washing, the IPs were electrophoresed in 8% SDS-PAGE gels and transferred to nitrocellulose membranes. Immunoblotting of the IPs was performed using a mouse monoclonal anti–TIF1-γ antibody or a rabbit polyclonal anti-CCAR1 antibody (for IPs performed with anti-CCAR1 or anti–TIF1-γ, respectively) as described above.

### Statistics.

All analyses were performed using Stata version 14. Logistic regression and Fischer’s exact testing were used to assess associations between CCAR1 autoantibodies and cancer. Differences between continuous variables were summarized and significance analyzed using a *t* test or Mann-Whitney test (normally vs. not normally distributed variables, respectively).

### Study approval.

The Stanford and Johns Hopkins IRBs approved the protocol for collection of plasma/serum from the DM patients in this study. All patients provided written informed consent before inclusion in the study.

## Author contributions

DFF, AR, and LCR designed the research study. DFF, CAM, LCS, AR, and LCR collected and clinically annotated patient sera and performed blinded visual inspection of the IP data. LCR and AR conducted experiments and were responsible for assigning positive/negative antibody status. CAM and MCR performed statistical data analysis. DFF, CAM, MCR, AR, and LCR performed general data analysis and interpretation. DFF, CAM, AR, and LCR wrote the manuscript. The order of first authorship was determined by extent of involvement in original research study design and analysis of the initial data.

## Supplementary Material

Supplemental data

ICMJE disclosure forms

## Figures and Tables

**Table 6:**
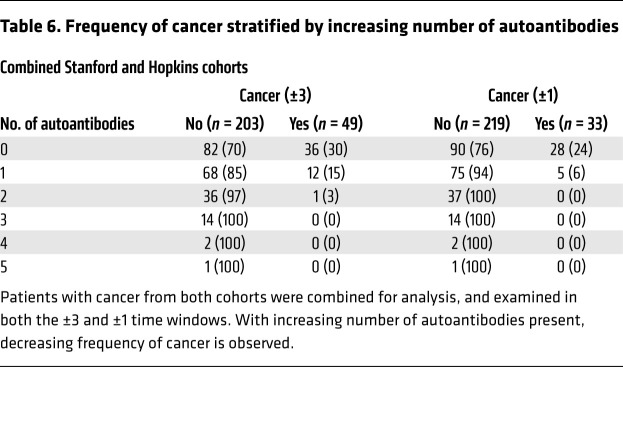
Frequency of cancer stratified by increasing number of autoantibodies

**Table 5:**
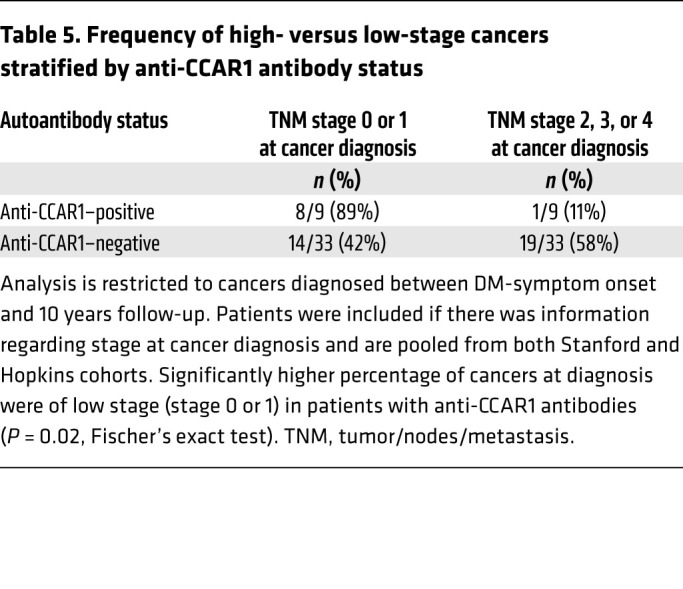
Frequency of high- versus low-stage cancers stratified by anti-CCAR1 antibody status

**Table 4:**
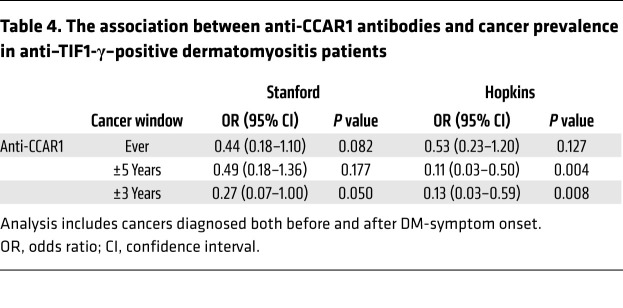
The association between anti-CCAR1 antibodies and cancer prevalence in anti–TIF1-γ–positive dermatomyositis patients

**Table 3:**
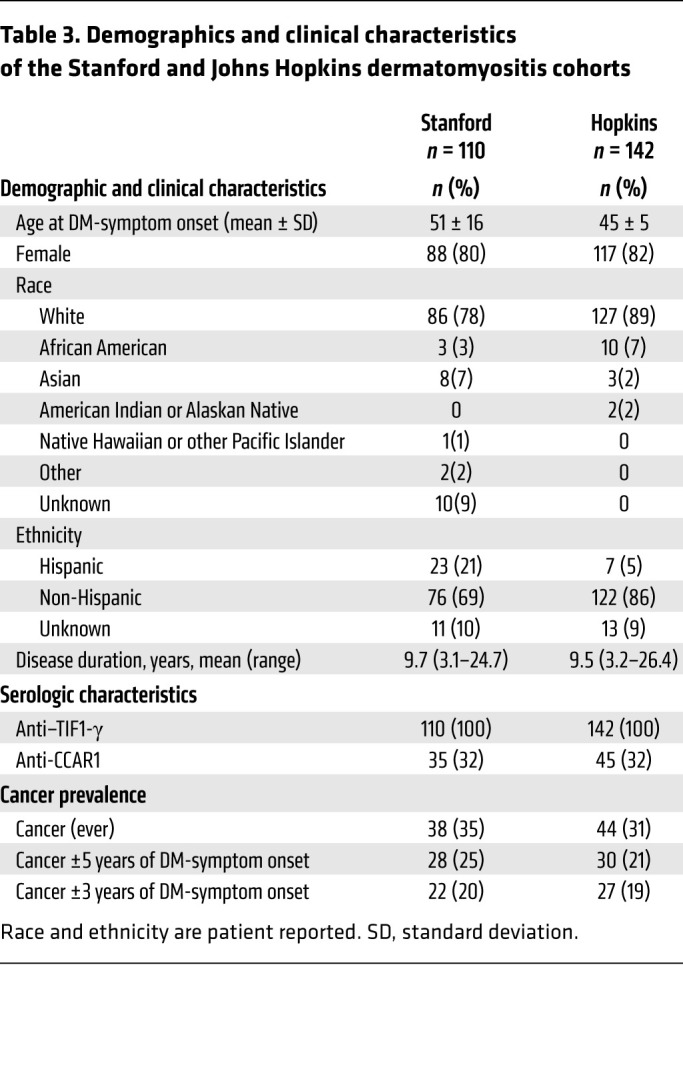
Demographics and clinical characteristics of the Stanford and Johns Hopkins dermatomyositis cohorts

**Table 2:**
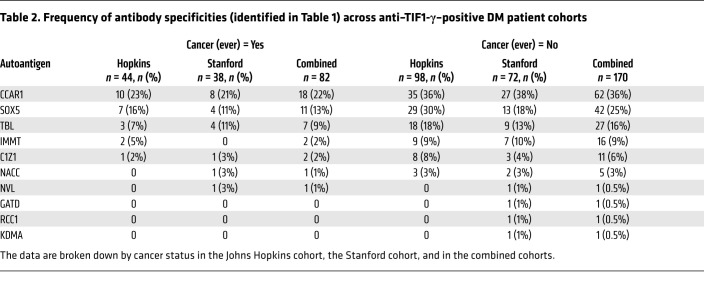
Frequency of antibody specificities (identified in Table 1) across anti–TIF1-γ–positive DM patient cohorts

**Table 1:**
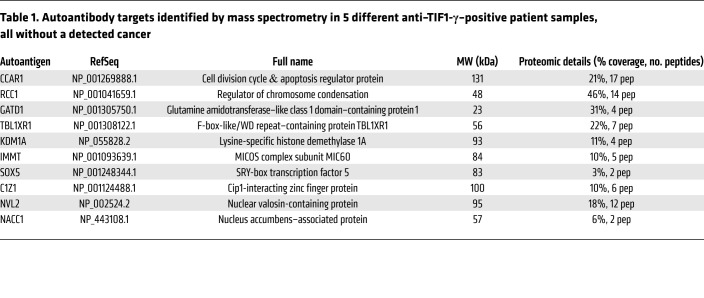
Autoantibody targets identified by mass spectrometry in 5 different anti–TIF1-γ–positive patient samples, all without a detected cancer

## Version 1 01/18/2022

Electronic publication
