# Supplementary material for: Immune responses to CCAR1 and other dermatomyositis autoantigens are associated with attenuated cancer emergence
Source: J Clin Invest. 2022 Jan 18;132(2):e150201. doi: 10.1172/JCI150201 (PMC8759791; doi:10.1172/JCI150201)
Supplement: Supplemental data [file jci-132-150201-s157.pdf]

SUPPLEMENTARY INFORMATION

Table S1. List of candidate hits not followed up for validation

Autoantigen	RefSeq	Full name	Mwt (kDa)
PPP1R21	NP_001129101.1	Protein phosphatase 1 regulatory subunit 21	88
CDC2	NP_001307847.1	Cyclin-dependent kinase 1	34
SMCHD1	NP_056110.2	Structural maintenance of chromosomes flexible hinge domain-containing protein 1	226
EML3	NP_694997.2	Echinoderm microtubule-associated protein-like 3	95
PRR4	NP_009175.2	Proline-rich protein 4	15
KCTD3	NP_057205.2	BTB/POZ Domain-Containing Protein KCTD3	89
BAX	NP_001278357.1	Apoptosis regulator BAX	24
TAF4	NP_003176.2	Transcription initiation factor TFIID subunit 4	110
TBCK	NP_001156907.1	TBC domain-containing	101
PHB2	NP_001138303.1	Prohibitin-2	33

Table S2. The effect of anti-CCAR1 on cancer prevalence in anti-TIF1-γ-positive dermatomyositis patients. Analysis includes cancers diagnosed only after DM-symptom onset.

		Stanford		Hopkins	
	Cancer Window	OR (95% CI)	p-value	OR (95% CI)	p-value
CCAR1	Ever	**0.28 (0.08-0.89)**	**0.031**	0.54 (0.20-1.47)	0.228
	Within 5 Years	0.45 (0.14-1.48)	0.190	**0.09 (0.01-0.70)**	**0.022**
	Within 3 Years	**0.12 (0.02-0.95)**	**0.045**	**0.09 (0.01-0.76)**	**0.026**

Table S3. Full list of all 82 cancers from both Johns Hopkins and Stanford Cohorts. Cancer type, timing, stage and autoantibody status of individual patients. NHL, non-Hodgkin's lymphoma; SCC, squamous cell carcinoma. "." Indicates data not available.

Cancer Type	Time from Cancer to DM Onset (years)	Cancer Stage Upon Diagnosis	Autoantibody Status
Prostate	-13.2	.	CCAR1
Breast	-12.4	.	CCAR1
Leukemia	-10.96	.	CCAR1
Breast	-10.9	1	CCAR1
Lung	-6.96	3	CCAR1
Melanoma	-6.03	0	CCAR1
Breast	-0.917	2	CCAR1
Breast	-0.88	2	CCAR1
Melanoma	0	0	CCAR1
Uterus	2.06	1	CCAR1
Breast	2.46	0	CCAR1
Kidney	3.48	1	CCAR1
Uterus	3.74	1	CCAR1
Melanoma	4.89	1	CCAR1
Breast	5.36	2	CCAR1
Lung and Bronchus	6.33	1	CCAR1
Breast	6.39	0	CCAR1
Lung	8.05	.	CCAR1
Thyroid	12.96	3	CCAR1
Breast	-17.08	.	Negative
Uterus	-16.9	.	Negative
Breast	-15.3	.	Negative
Breast	-4.97	1	Negative
Prostate	-4.53	2	Negative
Colon	-4.09	3	Negative
Breast	-2.66	2	Negative
Thyroid	-2.2	3	Negative
Uterus	-1.99	.	Negative
NHL	-1.91	3	Negative
NHL	-0.96	.	Negative
Ovary	-0.94	4	Negative
Breast	-0.721	0	Negative
Ovary	-0.547	3	Negative
Breast	-0.38	4	Negative
Ovary	-0.281	3	Negative
Melanoma	-0.235	0	Negative

Esophagus	-0.172	3	Negative
Prostate	-0.12	2	Negative
Lung	0.0136	4	Negative
Breast	0.076	1	Negative
Breast	0.136	1	Negative
Nasopharyngeal	0.167	.	Negative
Ovary	0.199	4	Negative
Uterus	0.251	3	Negative
Lung	0.257	2	Negative
Bladder	0.312	2	Negative
Breast	0.328	2	Negative
NHL	0.407	1	Negative
Kidney	0.443	.	Negative
Breast	0.449	1	Negative
Colon	0.563	2	Negative
Ovary	0.569	3	Negative
Breast	0.662	2	Negative
Breast	0.676	1	Negative
Melanoma	0.728	0	Negative
Thyroid	0.747	.	Negative
Colon	0.788	1	Negative
Colon	0.845	2	Negative
Skin (NHL)	0.9	.	Negative
Hodgkin's Lymphoma	0.958	2	Negative
Breast	1.33	0	Negative
Hodgkin's Lymphoma	1.46	2	Negative
Colon	1.49	1	Negative
Esophagus	1.579	.	Negative
Colon	1.61	4	Negative
Uterus	2.04	4	Negative
Breast	2.12	2	Negative
Breast	2.19	4	Negative
Thyroid	2.41	.	Negative
Desmoid Tumor	2.87	0	Negative
NHL	3.16	2	Negative
Oropharynx/SCC	3.19	1	Negative
Breast	4.2	0	Negative
Multiple Myeloma	6.79	1	Negative
Breast	7.24	0	Negative
Ovary	9.34	4	Negative
Oropharynx/SCC	9.53	4	Negative
Lung	10.06	2	Negative

Uterus	10.3	1	Negative
Prostate	11.2	2	Negative
Breast	16	2	Negative
Breast	18.65	1	Negative

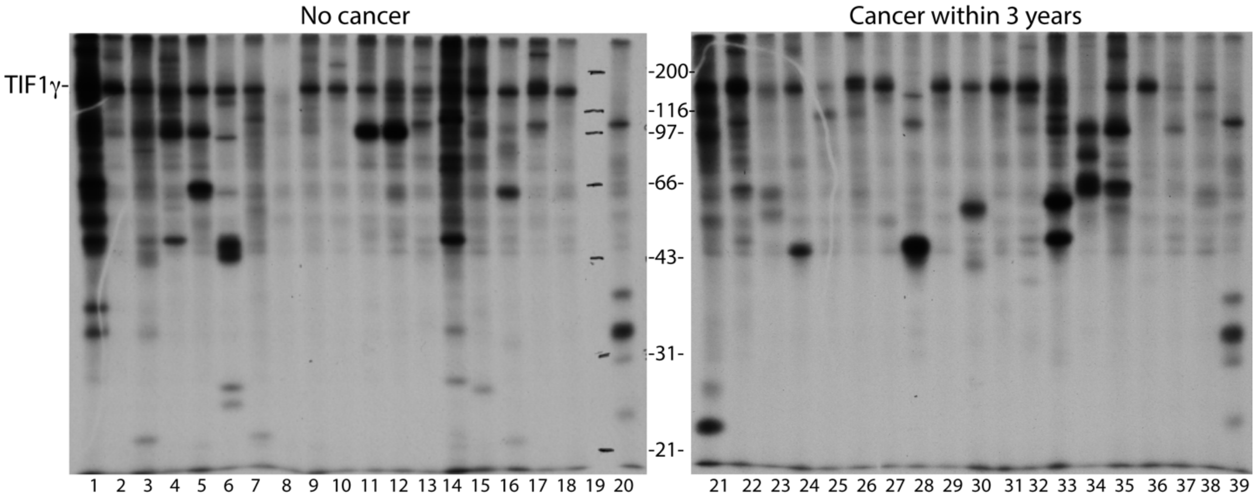

Fig. S1. Immunoprecipitations performed using radiolabeled cell lysates. 624 Melanoma cells were radiolabeled with ^{35}S-methionine and used for immunoprecipitations performed with plasma from 36 anti-TIF1-γ-positive dermatomyositis patients. Samples were from patients without (left panel) or with (right panel) a cancer detected within 3 years of dermatomyositis diagnosis. In each set, an IP performed with the same anti-PMSCL-positive patient serum (lanes 20 and 39) was included. This was done to facilitate equivalent exposures of the fluorograms based on the reference IP band intensities. Migration of molecular weight standards are marked between the panels, and are shown in lane 19.

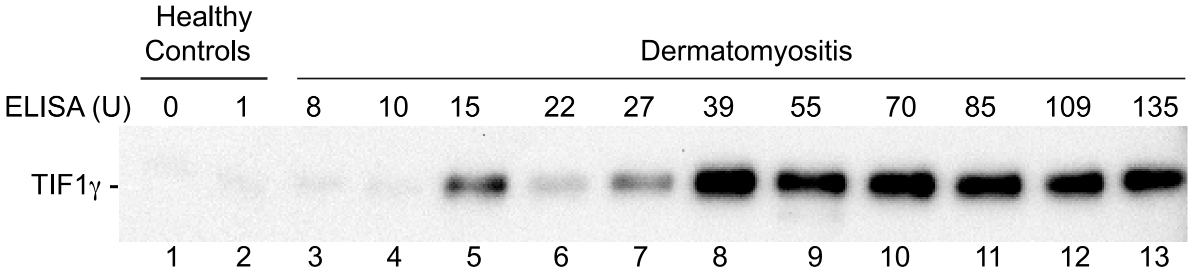

A. Comparison of ELISA and IP/blot assays to detect TIF1γ antibodies

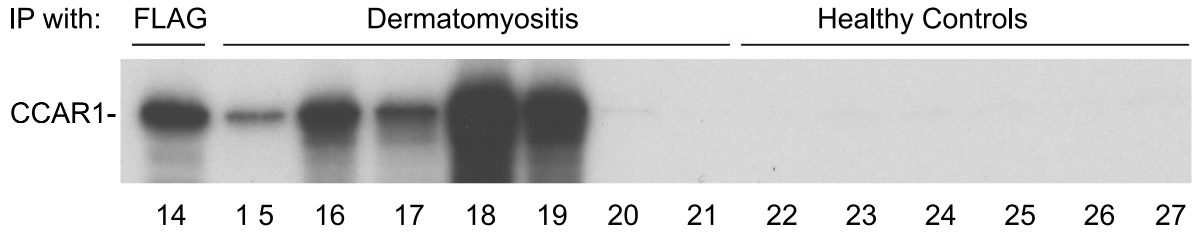

B. IVTTIPs to detect CCAR1 antibodies

Fig. S2. Assays to detect antibodies against TIF1-γ and CCAR1. (**A**) A comparison of ELISA and IP/blot assays to detect anti-TIF1-γ antibodies. Immunoprecipitations (IPs) were performed using TIF1-*γ*–transfected lysates and plasma from 11 anti-TIF1-γ positive dermatomyositis patients (lanes 3-13) or from 2 healthy controls (lanes 1 & 2) as described(*7*). The IPs were detected by blotting with an anti-TIF-1*γ* monoclonal antibody. The same samples were also tested using a commercially available ELISA assay to determine anti-TIF1-γ antibodies; results of this assay (units) are noted above the IP panel. (**B**) IP assay to identify patients with anti-cell division cycle and apoptosis regulator protein (CCAR1) antibodies. Plasma samples from 7 anti-TIF1-γ-positive dermatomyositis patients (lanes 15-21) and 6 healthy controls (lanes 22-27) were used to immunoprecipitate ^{35}S-methionine-labeled CCAR1 generated by in vitro transcription/translation. Dermatomyositis samples 15-19 are anti-CCAR1 positive, 20 & 21 are anti-CCAR1 negative, as are the controls. An anti-FLAG calibrator IP, performed using an anti-FLAG monoclonal antibody (Sigma), is shown in lane 14.

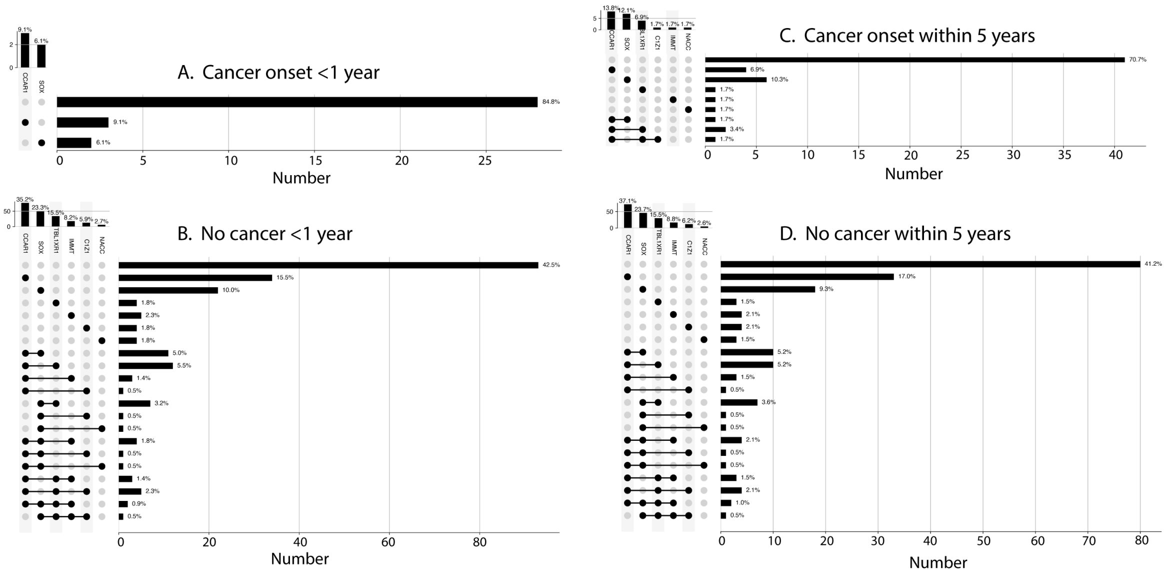

Fig. S3. Protection against cancer is associated with combinatorial expression of autoantibodies. Number, identity, and frequency of unique autoantibody combinations in patients with (A) or without (B) cancer within 1 year of DM onset, or with (C) or without (D) cancer within 5 years of DM onset. The vertical histogram above the matrix shows the frequency of specific autoantibodies in the cohort of anti-TIF1-g-positive patients, in order of decreasing magnitude. The y axis of those plots denotes number of patients. In the matrix itself, each row represents one autoantibody combination. Grey circles denote absence of a specific antibody, black circles denote presence, and when multiple specificities are present in a combination, they are connected by black lines. The frequency of each combination is shown in the horizontal barplots; the X-axis denotes the number of patients.
