# Supplementary material for: Immune responses to CCAR1 and other dermatomyositis autoantigens are associated with attenuated cancer emergence
Source: J Clin Invest. 2022 Jan 18;132(2):e150201. doi: 10.1172/JCI150201 (PMC8759791; doi:10.1172/JCI150201)
Supplement: ICMJE disclosure forms [file jci-132-150201-s158.pdf]

ICMJE DISCLOSURE FORM

Date:_____9/9/2021____
Your Name:____David Fiorentino____
Manuscript Title: Immune responses to CCAR1 and other novel dermatomyositis autoantigens are associated with attenuated cancer emergence.
Manuscript number: 15201-JCI-RG-1

In the interest of transparency, we ask you to disclose all relationships/activities/interests listed below that are related to the content of your manuscript. "Related" means any relation with for-profit or not-for-profit third parties whose interests may be affected by the content of the manuscript. Disclosure represents a commitment to transparency and does not necessarily indicate a bias. If you are in doubt about whether to list a relationship/activity/interest, it is preferable that you do so.

The following questions apply to the author's relationships/activities/interests as they relate to the current manuscript only.

The author's relationships/activities/interests should be defined broadly. For example, if your manuscript pertains to the epidemiology of hypertension, you should declare all relationships with manufacturers of antihypertensive medication, even if that medication is not mentioned in the manuscript.

In item #1 below, report all support for the work reported in this manuscript without time limit. For all other items, the time frame for disclosure is the past 36 months.

		**Name all entities with whom you have this relationship or indicate none (add rows as needed)**	**Specifications/Comments (e.g., if payments were made to you or to your institution)**
**Time frame: Since the initial planning of the work**			
1	All support for the present manuscript (e.g., funding, provision of study materials, medical writing, article processing charges, etc.) **No time limit for this item.**	____ None	
**Time frame: past 36 months**			
2	Grants or contracts from any entity (if not indicated in item #1 above).		
		Pfizer	Payment to institution
3	Royalties or licenses	____ None	

4	Consulting fees	Pfizer	Payments to me
		Corbus	Payments to me
5	Payment or honoraria for lectures, presentations, speakers bureaus, manuscript writing or educational events	none	
6	Payment for expert testimony	____ None	
7	Support for attending meetings and/or travel	____ None	
8	Patents planned, issued or pending	____ None	
9	Participation on a Data Safety Monitoring Board or Advisory Board	____ None	
10	Leadership or fiduciary role in other board, society, committee or advocacy group, paid or unpaid	____ None	
11	Stock or stock options	____ None	
12	Receipt of equipment, materials, drugs, medical writing, gifts or other services	____ None	
13	Other financial or non-financial interests	____ None	

Please place an "X" next to the following statement to indicate your agreement:

x I certify that I have answered every question and have not altered the wording of any of the questions on this form.

ICMJE DISCLOSURE FORM

Date: ___9/9/2021____
Your Name: ______Christopher A Mecoli____
Manuscript Title: Immune responses to CCAR1 and other novel dermatomyositis autoantigens are associated with attenuated cancer emergence.
Manuscript number: 15201-JCI-RG-1

In the interest of transparency, we ask you to disclose all relationships/activities/interests listed below that are related to the content of your manuscript. "Related" means any relation with for-profit or not-for-profit third parties whose interests may be affected by the content of the manuscript. Disclosure represents a commitment to transparency and does not necessarily indicate a bias. If you are in doubt about whether to list a relationship/activity/interest, it is preferable that you do so.

The following questions apply to the author's relationships/activities/interests as they relate to the current manuscript only.

The author's relationships/activities/interests should be defined broadly. For example, if your manuscript pertains to the epidemiology of hypertension, you should declare all relationships with manufacturers of antihypertensive medication, even if that medication is not mentioned in the manuscript.

In item #1 below, report all support for the work reported in this manuscript without time limit. For all other items, the time frame for disclosure is the past 36 months.

		**Name all entities with whom you have this relationship or indicate none (add rows as needed)**	**Specifications/Comments (e.g., if payments were made to you or to your institution)**
		**Time frame: Since the initial planning of the work**	
1	All support for the present manuscript (e.g., funding, provision of study materials, medical writing, article processing charges, etc.) **No time limit for this item.**	NIAMS Grant K23AR075898, Jerome L Greene Foundation	
		**Time frame: past 36 months**	
2	Grants or contracts from any entity (if not indicated in item #1 above).	____ None	
3	Royalties or licenses	____ None	

4	Consulting fees	____ None	
5	Payment or honoraria for lectures, presentations, speakers bureaus, manuscript writing or educational events	____ None	
6	Payment for expert testimony	____ None	
7	Support for attending meetings and/or travel	____ None	
8	Patents planned, issued or pending	____ None	
9	Participation on a Data Safety Monitoring Board or Advisory Board	____ None	
10	Leadership or fiduciary role in other board, society, committee or advocacy group, paid or unpaid	____ None	
11	Stock or stock options	____ None	
12	Receipt of equipment, materials, drugs, medical writing, gifts or other services	____ None	
13	Other financial or non-financial interests	____ None	

Please place an "X" next to the following statement to indicate your agreement:

_X__ I certify that I have answered every question and have not altered the wording of any of the questions on this form.

ICMJE DISCLOSURE FORM

Date:_09/09/2021____
Your Name:_Matthew Rosen____
Manuscript Title: Immune responses to CCAR1 and other novel dermatomyositis autoantigens are associated with attenuated cancer emergence.
Manuscript number: 15201-JCI-RG-1

In the interest of transparency, we ask you to disclose all relationships/activities/interests listed below that are related to the content of your manuscript. "Related" means any relation with for-profit or not-for-profit third parties whose interests may be affected by the content of the manuscript. Disclosure represents a commitment to transparency and does not necessarily indicate a bias. If you are in doubt about whether to list a relationship/activity/interest, it is preferable that you do so.

The following questions apply to the author's relationships/activities/interests as they relate to the current manuscript only.

The author's relationships/activities/interests should be defined broadly. For example, if your manuscript pertains to the epidemiology of hypertension, you should declare all relationships with manufacturers of antihypertensive medication, even if that medication is not mentioned in the manuscript.

In item #1 below, report all support for the work reported in this manuscript without time limit. For all other items, the time frame for disclosure is the past 36 months.

		**Name all entities with whom you have this relationship or indicate none (add rows as needed)**	**Specifications/Comments (e.g., if payments were made to you or to your institution)**
		**Time frame: Since the initial planning of the work**	
1	All support for the present manuscript (e.g., funding, provision of study materials, medical writing, article processing charges, etc.) **No time limit for this item.**	__X__ None	
		**Time frame: past 36 months**	
2	Grants or contracts from any entity (if not indicated in item #1 above).	__X__ None	
3	Royalties or licenses	__X__ None	

4	Consulting fees	__X__ None	
5	Payment or honoraria for lectures, presentations, speakers bureaus, manuscript writing or educational events	__X__ None	
6	Payment for expert testimony	__X__ None	
7	Support for attending meetings and/or travel	__X__ None	
8	Patents planned, issued or pending	__X__ None	
9	Participation on a Data Safety Monitoring Board or Advisory Board	__X__ None	
10	Leadership or fiduciary role in other board, society, committee or advocacy group, paid or unpaid	__X__ None	
11	Stock or stock options	__X__ None	
12	Receipt of equipment, materials, drugs, medical writing, gifts or other services	__X__ None	
13	Other financial or non-financial interests	__X__ None	

Please place an "X" next to the following statement to indicate your agreement:

__X__ I certify that I have answered every question and have not altered the wording of any of the questions on this form.

ICMJE DISCLOSURE FORM

Date: 9/9/21
Your Name:__ Lisa Christopher-Stine
Manuscript Title: Immune responses to CCAR1 and other novel dermatomyositis autoantigens are associated with attenuated cancer emergence.
Manuscript number (if known) 15201-JCI-RG-1

In the interest of transparency, we ask you to disclose all relationships/activities/interests listed below that are related to the content of your manuscript. "Related" means any relation with for-profit or not-for-profit third parties whose interests may be affected by the content of the manuscript. Disclosure represents a commitment to transparency and does not necessarily indicate a bias. If you are in doubt about whether to list a relationship/activity/interest, it is preferable that you do so.

The following questions apply to the author's relationships/activities/interests as they relate to the current manuscript only.

The author's relationships/activities/interests should be defined broadly. For example, if your manuscript pertains to the epidemiology of hypertension, you should declare all relationships with manufacturers of antihypertensive medication, even if that medication is not mentioned in the manuscript.

In item #1 below, report all support for the work reported in this manuscript without time limit. For all other items, the time frame for disclosure is the past 36 months.

		**Name all entities with whom you have this relationship or indicate none (add rows as needed)**	**Specifications/Comments (e.g., if payments were made to you or to your institution)**
		**Time frame: Since the initial planning of the work**	
1	All support for the present manuscript (e.g., funding, provision of study materials, medical writing, article processing charges, etc.) **No time limit for this item.**	_x___None	
		**Time frame: past 36 months**	
2	Grants or contracts from any entity (if not indicated in item #1 above).	Pfizer	Paid to the institution; clinical trial support
		Corbus	Paid to the institution; clinical trial support
		Kezar	Paid to the institution; clinical trial support
3	Royalties or licenses	Inova Diagnostics	Royalties for IP related to anti-HMGCR assay
4	Consulting fees	Janssen	Consultant; paid to me
		Boehringer-Ingelheim	Consultant; paid to me
		Mallinckrodt	Consultant; paid to me

		EMD-Serono	Consultant; paid to me
		ArgenX	Consultant; paid to me
		Allogene	Consultant; paid to me
		Octapharma	Consultant; paid to me
		Roivant	Consultant; paid to me
5	Payment or honoraria for lectures, presentations, speakers bureaus, manuscript writing or educational events	_x___None	
6	Payment for expert testimony	Bendin Sumrall and Ladner LLC	
		Feldman, Kleidman Coffey & Sappe LLP	
		Downs Ward Bender Hauptmann & Herzog, P.A.	
		Sulloway and Hollis	
7	Support for attending meetings and/or travel	_x___None	
8	Patents planned, issued or pending	Inova Diagnostics/RDL	Anti-HMGCR assay
9	Participation on a Data Safety Monitoring Board or Advisory Board	_x___None	
10	Leadership or fiduciary role in other board, society, committee or advocacy group, paid or unpaid	__x__None	
11	Stock or stock options	x____None	
12	Receipt of equipment, materials, drugs, medical writing, gifts or other services	x____None	
13	Other financial or non-financial interests	__x__None	

Please place an "X" next to the following statement to indicate your agreement:

__x_ I certify that I have answered every question and have not altered the wording of any of the questions on this form.

ICMJE DISCLOSURE FORM

Date:______09/09/2021____
Your Name:_____Lorinda Chung____
Manuscript Title: Immune responses to CCAR1 and other novel dermatomyositis autoantigens are associated with attenuated cancer emergence.
Manuscript number: 15201-JCI-RG-1

In the interest of transparency, we ask you to disclose all relationships/activities/interests listed below that are related to the content of your manuscript. "Related" means any relation with for-profit or not-for-profit third parties whose interests may be affected by the content of the manuscript. Disclosure represents a commitment to transparency and does not necessarily indicate a bias. If you are in doubt about whether to list a relationship/activity/interest, it is preferable that you do so.

The following questions apply to the author's relationships/activities/interests as they relate to the current manuscript only.

The author's relationships/activities/interests should be defined broadly. For example, if your manuscript pertains to the epidemiology of hypertension, you should declare all relationships with manufacturers of antihypertensive medication, even if that medication is not mentioned in the manuscript.

In item #1 below, report all support for the work reported in this manuscript without time limit. For all other items, the time frame for disclosure is the past 36 months.

		Name all entities with whom you have this relationship or indicate none (add rows as needed)	Specifications/Comments (e.g., if payments were made to you or to your institution)
		**Time frame: Since the initial planning of the work**	
1	All support for the present manuscript (e.g., funding, provision of study materials, medical writing, article processing charges, etc.) **No time limit for this item.**	__X__ None	
		**Time frame: past 36 months**	
2	Grants or contracts from any entity (if not indicated in item #1 above).	____ None	Grant funding from Boerhinger Ingelheim
3	Royalties or licenses	____ None	

4	Consulting fees	____ None	Consulting fees from Boerhinger Ingelheim, Eicos Sciences, Genentech, Kyverna, Mitsubishi Tanabe
5	Payment or honoraria for lectures, presentations, speakers bureaus, manuscript writing or educational events	____ None	
6	Payment for expert testimony	____ None	
7	Support for attending meetings and/or travel	____ None	
8	Patents planned, issued or pending	____ None	
9	Participation on a Data Safety Monitoring Board or Advisory Board	____ None	
10	Leadership or fiduciary role in other board, society, committee or advocacy group, paid or unpaid	____ None	
11	Stock or stock options	____ None	
12	Receipt of equipment, materials, drugs, medical writing, gifts or other services	____ None	
13	Other financial or non-financial interests	____ None	

Please place an "X" next to the following statement to indicate your agreement:

_X__ I certify that I have answered every question and have not altered the wording of any of the questions on this form.

ICMJE DISCLOSURE FORM

Date:_____September 12, 2020____
Your Name:_______Antony Rosen____
Manuscript Title: Immune responses to CCAR1 and other novel dermatomyositis autoantigens are associated with attenuated cancer emergence.
Manuscript number: 15201-JCI-RG-1

In the interest of transparency, we ask you to disclose all relationships/activities/interests listed below that are related to the content of your manuscript. "Related" means any relation with for-profit or not-for-profit third parties whose interests may be affected by the content of the manuscript. Disclosure represents a commitment to transparency and does not necessarily indicate a bias. If you are in doubt about whether to list a relationship/activity/interest, it is preferable that you do so.

The following questions apply to the author's relationships/activities/interests as they relate to the current manuscript only.

The author's relationships/activities/interests should be defined broadly. For example, if your manuscript pertains to the epidemiology of hypertension, you should declare all relationships with manufacturers of antihypertensive medication, even if that medication is not mentioned in the manuscript.

In item #1 below, report all support for the work reported in this manuscript without time limit. For all other items, the time frame for disclosure is the past 36 months.

		**Name all entities with whom you have this relationship or indicate none (add rows as needed)**	**Specifications/Comments (e.g., if payments were made to you or to your institution)**
**Time frame: Since the initial planning of the work**			
1	All support for the present manuscript (e.g., funding, provision of study materials, medical writing, article processing charges, etc.) **No time limit for this item.**	NIH grants RO1AR073208 and P30-AR070254, and the Stabler Foundation	Payments made to Johns Hopkins
**Time frame: past 36 months**			
2	Grants or contracts from any entity (if not indicated in item #1 above).	_____	
3	Royalties or licenses	Inova Diagnostics	Royalty payments for several autoantibody assays (HMGCR, PAD4), not related to this manuscript
		Celgene	Royalty payments for PAD4 assay (unrelated to this manuscript)

4	Consulting fees	____ None	
5	Payment or honoraria for lectures, presentations, speakers bureaus, manuscript writing or educational events	____ None	
6	Payment for expert testimony	____ None	
7	Support for attending meetings and/or travel	____ None	
8	Patents planned, issued or pending	____ Patent application pending	
9	Participation on a Data Safety Monitoring Board or Advisory Board	____ None	
10	Leadership or fiduciary role in other board, society, committee or advocacy group, paid or unpaid	____ None	
11	Stock or stock options	____ None	
12	Receipt of equipment, materials, drugs, medical writing, gifts or other services	____ None	
13	Other financial or non-financial interests	____ None	

Please place an "X" next to the following statement to indicate your agreement:

_X__ I certify that I have answered every question and have not altered the wording of any of the questions on this form.

ICMJE DISCLOSURE FORM

Date: 09/09/2021____
Your Name:_ Livia Casciola-Rosen____
Manuscript Title: Immune responses to CCAR1 and other novel dermatomyositis autoantigens are associated with attenuated cancer emergence.
Manuscript number: 15201-JCI-RG-1

In the interest of transparency, we ask you to disclose all relationships/activities/interests listed below that are related to the content of your manuscript. "Related" means any relation with for-profit or not-for-profit third parties whose interests may be affected by the content of the manuscript. Disclosure represents a commitment to transparency and does not necessarily indicate a bias. If you are in doubt about whether to list a relationship/activity/interest, it is preferable that you do so.

The following questions apply to the author's relationships/activities/interests as they relate to the current manuscript only.

The author's relationships/activities/interests should be defined broadly. For example, if your manuscript pertains to the epidemiology of hypertension, you should declare all relationships with manufacturers of antihypertensive medication, even if that medication is not mentioned in the manuscript.

In item #1 below, report all support for the work reported in this manuscript without time limit. For all other items, the time frame for disclosure is the past 36 months.

		**Name all entities with whom you have this relationship or indicate none (add rows as needed)**	**Specifications/Comments (e.g., if payments were made to you or to your institution)**
		**Time frame: Since the initial planning of the work**	
1	All support for the present manuscript (e.g., funding, provision of study materials, medical writing, article processing charges, etc.) **No time limit for this item.**	NIH grants RO1AR073208 and P30-AR070254, and the Stabler Foundation	
		**Time frame: past 36 months**	
2	Grants or contracts from any entity (if not indicated in item #1 above).	____ None	
3	Royalties or licenses	Inova Diagnostics	Royalties for IP related to anti-HMGCR assay

4	Consulting fees	____ None	
5	Payment or honoraria for lectures, presentations, speakers bureaus, manuscript writing or educational events	____ None	
6	Payment for expert testimony	____ None	
7	Support for attending meetings and/or travel	____ None	
8	Patents planned, issued or pending	Patent planned: "Methods & Materials for treating cancer"	
9	Participation on a Data Safety Monitoring Board or Advisory Board	____ None	
10	Leadership or fiduciary role in other board, society, committee or advocacy group, paid or unpaid	____ None	
11	Stock or stock options	____ None	
12	Receipt of equipment, materials, drugs, medical writing, gifts or other services	____ None	
13	Other financial or non-financial interests	____ None	

Please place an "X" next to the following statement to indicate your agreement:

X I certify that I have answered every question and have not altered the wording of any of the questions on this form.
